# The role of trauma, attachment, and voice-hearer’s appraisals: a latent profile analysis in the AVATAR2 trial

**DOI:** 10.1017/S003329172500008X

**Published:** 2025-02-27

**Authors:** Julia Marotti, Rob Saunders, Alice Montague, Miriam Fornells-Ambrojo

**Affiliations:** 1 Research Department of Clinical, Educational and Health Psychology, University College London, London, UK; 2 CORE Data Lab, Centre for Outcomes Research and Effectiveness (CORE), Research Department of Clinical, Educational and Health Psychology, University College London, London, UK; 3 North East London NHS Foundation Trust, London, UK

**Keywords:** Auditory verbal hallucination, Psychosis, Trauma, Attachment, Voice appraisals, Latent Profile Analysis, AVATAR therapy

## Abstract

**Background:**

There is evidence that attachment, trauma, and voice appraisals individually impact voice hearing in psychosis, but their intersectional relationship has not been examined. The aim of this study was to identify subgroups of individuals from the intersectional relationship between these factors and examine differences between subgroups on clinical outcomes.

**Methods:**

A latent profile analysis was conducted on baseline data from the AVATAR2 trial (*n* = 345), to identify statistically distinct subgroups of individuals with psychosis who hear distressing voices based on co-occurring patterns of trauma, fearful attachment, and voice appraisals. The association between profile membership and demographic characteristics, voice severity, posttraumatic stress disorder symptoms, emotional distress, and difficulties with motivation and pleasure was then examined. Experts by experience were consulted throughout the process.

**Results:**

Four profiles were identified: ‘adverse voices and relational trauma’, ‘low malevolent and omnipotent voices’, ‘adverse voices yet low relational trauma’, and ‘high benevolent voices’. Negative voice appraisals occurred in the presence of high and low trauma and attachment adversities. The first profile was associated with being female and/or other non-male genders and had worse voice severity and emotional distress. High adversities and worse emotional distress occurred in the presence of voice benevolence and engagement. Black and South Asian ethnicities were not associated with specific profiles.

**Conclusions:**

The identified profiles had negative and positive voice appraisals associated with higher and lower occurrence of adversities, and different clinical outcomes. These profiles could inform detailed case formulations that could tailor interventions for voice hearers.

## Introduction

Auditory verbal hallucination or voice hearing in the absence of a corresponding external stimuli, referred to as “voices” henceforth, is understood to be on a continuum (Linscott & van Os, 2010). It has a lifetime prevalence rate of up to one in ten individuals in the general population (Maijer et al., 2018) associated with reduced distress (Taylor & Murray, 2012), and it occurs in a range of mental health disorders including psychosis, where voices are usually reported as more severe and distressing and linked with increased clinical needs (Larøi, 2012) and need for care (Johns et al., 2014). Voice distress in psychosis has been linked with increased anxiety, depression, and a higher degree of negative voice content (Scott, Rossell, Toh, & Thomas, 2020b), and the presence of posttraumatic stress disorder (PTSD; de Bont et al., 2015).

Voices in psychosis have been associated with a history of different types of trauma (Bailey et al., 2018; Grindey & Bradshaw, 2022), with voice content at times directly reflecting trauma content (e.g., hearing the voice of a past abuser; van den Berg et al., 2023). Traumatic stress-induced changes (i.e., traumagenic neurodevelopmental model; Pruessner et al., 2017) are implicated in emotional memories being encoded without contextual information (Brewin & Burgess, 2014). Trauma memories might be experienced on a continuum of contextualized autobiographical memories and fragmented sensory experiences, such as voices, that are appraised as externally sourced when re-experienced (Hardy, 2017).

Disrupted care experiences during childhood, including neglect, abuse, and early losses, are involved in the development of fearful attachment (van Ijzendoorn et al., 1999), one of three types of insecure attachment patterns developed during early caregiving relationships (Ainsworth & Bell, 1981). A higher prevalence of fearful attachment has been observed in individuals with psychosis (Carr et al., 2018a), with significantly higher levels of hallucinations and voice distress (Bucci et al., 2017).

Cognitive models of psychosis propose that childhood relational trauma and insecure attachment styles renders individuals more vulnerable to negative interpretations of self and others (Garety et al., 2001; Scott, Rossell, Meyer, et al., 2020a). These interpretations, especially when originating from experiences of subordination and marginalization, influence hearers’ appraisals of voices as more malevolent, persecuting, powerful, critical and are related to greater voice distress (Birchwood et al., 2000; Larøi et al., 2019). In non-clinical hearers, voices are reported as predominantly benevolent and less distressing (Daalman et al., 2011).

Relational theories also implicate interpersonal frameworks and social world relationships in how the hearer responds to voices, influencing distress (Birchwood et al., 2004; Thomas et al., 2009). It is then important to consider social factors and their effect on voices experiences (e.g., culture-wide/culture−specific views of voices; Luhrmann et al., 2015), accounting also for how social factors’ associations to voices are attenuated by age, gender, and ethnicity (Bonoldi et al., 2013). Thus, relational therapies aim to target distress by focusing on such relationships, enabling the individual to state their needs and gain awareness (Craig et al., 2018; Hayward et al., 2011).

Most research to date has investigated the interplay between trauma, attachment, and voice appraisals in isolation or paired associations, with debates over the specificity (i.e., one process leads to voices) and equifinality (i.e., voices may occur via different processes from a variety of different initial conditions) of the multiple mechanisms and factors involved (Gibson et al., 2016). Such traditional variable-centered studies provide estimated parameters indicating how factors are related in all individuals, which are assumed to be drawn from a single population, without considering how such factors may have different interactions which could differ across subpopulations of individuals (Morin et al., 2017). Conversely, person-centered analyses are data-driven (e.g., Begemann et al., 2022), relaxing this assumption, and aimed to unearth multiple specific combinations of factors in cross-sectional data based on how they differ or are similar in traits and dimensions of interest (Saunders et al., 2020).

This study aimed to (1) use latent profile analysis to identify statistically distinct groups of individuals who hear distressing voices, based on the interplay between experiences of trauma, fearful attachment style and voice appraisals, and (2) explore whether identified profiles are differentially associated with demographic characteristics and clinical outcomes. Involvement of Experts by Experience was incorporated at all stages of the analysis, in line with recommendations in research (i.e., Corstens et al., 2014; National Institute for Health and Care Research [NIHR], 2012) and with the aim that chosen measures and interpretation of findings had more meaningful and real-world clinical applications.

## Methods

### Setting

The baseline assessment data from the AVATAR2 multisite (London, Manchester, Glasgow) randomized controlled trial (RCT; see Garety et al., 2021 for detailed description) testing the efficacy of two versions of AVATAR therapy (Craig et al., 2018; Leff et al., 2014) against treatment-as-usual was used for this study. The study was approved by London-Camberwell St Giles Research Ethics Committee (REC/HRA ethical approval 20/LO/0657) which included consent for participation in ancillary/additional studies that utilize anonymized data collected, such as the current study. The recruitment, data collection of assessment meetings, and data storage were carried out according to the AVATAR2 trial protocol (please see Garety et al., 2021, 2024 for further information). The baseline assessment meetings completed asked participants about their experiences of their voices, overall mood, and wellbeing via multiple measures.

### Participants

The 345 participants included in this study met the inclusion and exclusion criteria of the AVATAR2 RCT (for full details, see Garety et al. (2021) and Supplementary Material A).

### Measures

Table 1 displays measures, their descriptions, and psychometric information, for the indicator variables included in the LPA, and the distal variables in post-LPA analyses, which are demographic characteristics and clinical presentation outcomes. For further details on measures and participant characteristics, see Table 1 and Supplementary Material B.

**Table 1.** Table with indicator and distal variables included in the latent profile analysis and further analyses and their measure description and properties (see Supplementary Material B for further details)

LPA role	Variable	Type of variable	Description of measure including properties
Indicator variables	Fearful attachment	Continuous	Score on the fearful attachment item of the Relationship Scale Questionnaire (RSQ; Griffin & Bartholomew, 1994). A few studies show good validity and reliability properties for the full RSQ measure (Wongpakaran et al., 2021), including across cultures (Schmitt et al., 2004) and with good agreement with other dimensional measures of attachment
	Trauma		Score on the Mini Trauma and Life Events (Mini-TALE; Carr et al., 2018b) indicating presence of four traumatic events: physical, emotional, sexual abuse, and neglect. The TALE was recently designed as a psychosis-specific trauma measure useful for clinical practice, as such it has limited investigation of quality and warrants further investigation of its psychometric properties (Airey et al., 2023)
	Beliefs about trauma-voice content relatedness		Score on the Trauma Voice Associations Questionnaire (TVAQ; Woods et al., 2015). This is a novel measure, linked to other phenomenological surveys (Woods et al., 2015). It requires further research into its psychometric properties, albeit this measure is one of the few specific measures capturing beliefs about how trauma experiences link to voices (Tolmeijer et al., 2021)
	Voice appraisals – Malevolence subscale		Score on the Beliefs About Voices Revised Questionnaire (BAVQ-R; Chadwick et al., 2000) concerning experiencing voices as malevolent, omnipotent, benevolent, and experiencing resistance to or engagement with voices This measure is widely used in voice hearing literature, with acceptable psychometric properties (Andrew et al., 2008; Ratcliff et al., 2011)
	Voice appraisals – Omnipotence subscale		
	Voice appraisals – Resistance subscale		
	Voice appraisals – Benevolence subscale		
	Voice appraisals – Engagement subscale		
Distal variables: Clinical	Voice hearing severity	Continuous	core on the Psychotic Symptoms Rating Scale-Auditory Hallucinations (PSYRATS- H; Haddock et al., 1999) including domains of distress and frequency of voices for the total severity score This scale is widely used in the literature, with good psychometric properties established in psychosis (Drake et al., 2007; Haddock et al., 1999; Ratcliff et al., 2011)
	PTSD symptoms		Score on 6 PTSD items in the International Trauma Questionnaire (ITQ; Cloitre et al., 2018). Completed by participants reporting 1+ trauma(s) on the Mini-TALE There are satisfactory levels of psychometric properties of this measure reported in the literature (Cloitre et al., 2021; Hyland et al., 2017)
	Level of global distress		Score on the Depression, Anxiety and Stress Scale (DASS–21; Henry & Crawford, 2005) yielding three subscales of depression, anxiety and stress This is a well-used clinical tool (Ng et al., 2007) with high-quality internal consistency yet further research is warranted to consider the low quality of evidence for measure reliability (Lee et al., 2019)
	Level of motivation and pleasure difficulties		Score on the Motivation and Pleasure (MAP) subscale of the Clinical Assessment Interview for Negative Symptoms (CAINS; Kring et al., 2013) This measure has good properties established in the psychosis literature (Horan et al., 2011)
Distal: Demographic	Age	Continuous	Age of participant
	Gender	Categorial	Defined as ‘Male’, or ‘Female’ or ‘Other non-male’ genders (‘other’ including non-binary, other responses)
	Ethnicity background		Defined as ‘White’, ‘Black or Mixed Black’, ‘South Asian or Mixed South Asian’, ‘Any Other’

### Analysis

#### Latent profile analysis

LPA is a mixture modeling approach extending latent class analysis (Vermunt & Magidson, 2002) to include both continuous and categorical variables (Gibson, 1959). LPA was conducted using Mplus version 8 (Muthén & Muthén, 2017) evaluating whether there are distinct groups (latent profiles; LPs) of voice hearing individuals based on the observed individual response patterns on the indicator variables of appraisal of voices (as malevolent, omnipotent, benevolent, and voice engagement or resistance), the presence of fearful attachment style, traumatic experiences, and association of trauma-voice content. Further information can be found in Supplementary Material C about the selection of indicator variables and negotiating with model convergence constraints.

Model fit of the LPA models was compared using The Vuong-Lo-Medell-Rubin likelihood ratio test (VLMR-LRT; Lo et al., 2001), and Bootstrap likelihood ratio difference test (B-LRT; Nylund et al., 2007) alongside Akaike Information Criterion (AIC), Bayesian Information Criterion (BIC), and entropy-based criterion values (Geiser, 2013). FIML is used for systematically missing data in LPA. The full information about the model selection process can be found in Supplementary Material D.

#### Associations between profiles and both demographic information and clinical outcomes

Following the LPA, a series of analyses were performed to explore the associations between a) LPs and demographic variables (age, gender, ethnicity) and b) LPs and clinical presentation outcomes (voice severity, PTSD symptoms, level of global distress and motivation and pleasure difficulties).

To account for misclassification accuracy in LPA (Bakk et al., 2013; Clark & Muthén, 2009), multinomial logistic regression analyses via the bias-adjusted R3STEP method (Asparouhov & Muthén, 2014) was used, with the reference profile for associations in a) being the largest sample size. Specifically, the recommended Bolck-Croon-Hagenaars (BCH) procedure (Bolck et al., 2004) was employed, with demographic variables (age, gender, ethnicity) included as confounders in models in b) examining differences in clinical outcomes across profiles (McLarnon & O’Neill, 2018; Nylund-Gibson et al., 2019). The profile-specific regression intercepts yielded were compared in omnibus Wald Chi-square tests and pairwise *z*-tests that suggest how belonging to one profile is differentially associated with a clinical outcome beyond the demographics and other profiles (Clark & Muthén, 2009). Missing data on demographic and clinical variables were handled using multiple imputation in Mplus (Section 11.1 of Asparouhov & Muthén, 2021). For detailed information about these post-LPA analyses, see Supplementary Material E.

### Patient and public involvement

In line with the Hearing Voices Movement (Corstens et al., 2014) and the NIHR (2012) recommendations to involve people with lived experiences in research, patient and public involvement (PPI) has played a key role at all stages for co-production of the AVATAR2 trial, including design, recruitment of staff and participants, data collection, analysis, and dissemination. An active and creative group of people was established, comprising 20 members across all four sites, from diverse backgrounds, with lived experience of psychosis and recovery, and including carers. PPI co-production was continued in this study for decision-making processes and to ensure that interpretations of the complex findings were clinically useful and with real-world reflections.

First, one-to-one consultations were conducted with five PPI consultants at the pre-analysis stage. They provided feedback about the importance of the chosen indicator variables, the retention of measures (e.g., trauma-voice content association (i.e., TVAQ; Woods et al., 2015), and the voice appraisals measure (i.e., BAVQ-R (Chadwick et al., 2000) and not the Voices Acceptance and Action Scale (Shawyer et al., 2007), or Voice Power Differential Scale (Birchwood et al., 2000) and informed the meaningful inclusion of ethnicity and gender demographics (i.e., exploring their association with the LPA model).

Consultations were also completed post-LPA-analysis with four PPI consultants. This explored their insights and understanding of the identified LPA groupings and their indicator variables’ distributions. A qualitative analysis of the rich feedback was beyond the scope of this study; thus, PPI reflections were incorporated when discussing the results. For further detailed information and summary of consultations, see Supplementary Material F.

## Results

### Descriptive statistics

The full sample of 345 participants had an average age of 39.6 years old, with a higher percentage of male gender (61.4%), ‘White’ ethnicity (59.7%), followed by ‘Black or mixed Black’ ethnicity (20.9%), and the majority had a Schizophrenia or Schizoaffective diagnosis (87.2%). The full sample results, from the RSQ and Mini-TALE, found a high rate of reported interpersonal adversities of fearful attachment and trauma (only 5.8% reported no trauma) alongside negative voice appraisals (i.e., omnipotence, malevolence) and resistance to voices, with lower occurrence of benevolent voices and engagement. Such indicator variables were mostly weakly to moderately associated. As expected, participants had high voice severity and emotional distress, high levels of motivation and pleasure difficulties (albeit just below cutoff score indicative of negative symptoms; Li et al., 2018), and a PTSD dimensional score indicative of a middle level of symptom severity (*M* = 10.6 out of a total of 24). See full details of the full sample statistics in Supplementary Material G.

### Latent profile analysis

The VLMRT-LRT and B-LRT yielded significant *p*-values (*p* < 0.05), alongside decreases in the AIC and BIC values, when comparing successive models from a two-profile to a four-profile solution. At the five-profile model, the VLMR-LRT *p*-value increased to above 0.05 (*p* = 0.198), and the entropy value for the four-profile versus the five-profile model was slightly higher, therefore the four-profile solution was considered optimal for the data (see Supplementary Material D for model comparison statistics).

#### Latent profiles descriptions

Descriptive statistics for the full sample and distributions for each latent profile (LP) are displayed in Table 2 (presented graphically in Figure 4 in Supplementary Material H), with the description of each profile provided here:

1. LP1 (largest; approximately 44.5%, *n* = 157) is described as ‘**Adverse voices and relational trauma’** – In comparison with other profiles, individuals in this profile have the highest scores, which are higher as compared to the full sample, of fearful attachment style, trauma, and are more likely to believe that trauma and voices are related. Compared to the full sample, they report moderately higher on beliefs of voices being malevolent, omnipotent, and resisting voices, the highest scores comparative to other profiles. It has the lowest scores of benevolent voice appraisal and engagement, akin to LP3.
2. LP2 (second largest; approximately 25.2%, *n* = 84) is described as ‘**Low malevolent and omnipotent voices**’– Individuals in this profile are set apart by their scoring the lowest on all subscales relating to negative voice appraisals of omnipotence, malevolence and resistance to voices, compared to other profiles and the full sample. Compared to the full sample, individuals in this group have a marginally lower number of traumas experienced, traumas’ relatedness to voices and fearful attachment. Benevolent voice appraisal and engagement scores were low, similar to the full sample, however not as low as LP1 and LP3.
3. LP3 (second smallest; approximately 16.5%, *n* = 57) is described as ‘**Adverse voices yet low relational trauma**’ – Individuals in this profile are reporting the lowest scores across profiles for relational traumas. Compared to the full sample they have much lower experiences of fearful attachment, moderately a lower number of traumas reported, and where such traumas are not believed to be associated to voices heard. Although, such individuals are also scoring the second highest on subscales relating to omnipotent and malevolent voice appraisals, which is marginally higher than average full sample. Resistance to voices is similar to the high score in the full sample. Additionally, akin to LP1, they have a lower score of benevolent voice appraisals and engagement.
4. LP4 (smallest; approximately 13.8%, *n* = 47) is described as ‘**High benevolent voices’** – Individuals in this profile are set apart by their scoring very high as compared to the full sample and other profiles on benevolent voice appraisal and engagement with voices. Although, they are similar to the full sample, with a higher number of traumas, beliefs of voices being related to traumas and experiencing fearful attachment and higher scores of omnipotent voice appraisals. Scores of malevolence voice appraisals and resistance to voices were low, lower than the full sample scores.

**Table 2.** Full sample and latent profiles indicator variable distribution

		Full sample (*n* = 345)	LP1 ‘Adverse voices and relational trauma’ (44.5%, *n* = 157)	LP2 ‘Low malevolent and omnipotent voices’ (25.2%, *n* = 84)	LP3 ‘Adverse voices yet low relational trauma’ (16.5%, *n* = 57)	LP4 ‘High benevolent voices’ (13.8%, *n* = 47)
Indicator variables	Measure^a^ (range)	Mean (SD)	Mean (SD)	Mean (SD)	Mean (SD)	Mean (SD)
Fearful attachment	RSQ (1–7)	5.1 (1.9) [*n* = 341]	6.2 (1.0) [*n* = 156]	4.7 (1.5) [*n* = 83]	2.2 (1.2) [*n* = 55]	5.5 (1.5) [*n* = 47]
Trauma	Mini-TALE (0–4)	2.7 (1.2) [*n* = 345]	3.1 (0.9) [*n* = 157]	2.5 (1.1) [*n* = 84]	1.8 (1.4) [*n* = 57]	3.0 (1.1) [*n* = 47]
Beliefs about trauma-voice content relatedness	TVAQ (0–3)	1.9 (1.1) [*n* = 322]	2.2 (1.0) [*n* = 153]	1.4 (1.1) [*n* = 78]	1.3 (1.1) [*n* = 45]	2.2 (1.1) [*n* = 46]
Voice appraisal subscales	BAVQ-R: Malevolence (0–18)	11.5 (4.3) [*n* = 343]	14.0 (2.7) [*n* = 157]	6.2 (3.0) [*n* = 84]	12.7 (3.0) [*n* = 55]	11.0 (3.6) [*n* = 47]
Voice appraisal subscales	BAVQ-R: Omnipotence (0–18)	11.1 (3.6) [*n* = 341]	12.6 (2.9) [*n* = 157]	7.1 (2.6) [*n* = 84]	12.3 (2.7) [*n* = 54]	12.0 (3.3) [*n* = 46]
Voice appraisal subscales	BAVQ-R: Resistance (0–27)	19.8 (4.7) [*n* = 342]	22 (3.5) [*n* = 157]	16.7 (4.8) [*n* = 83]	19.4 (4.9) [*n* = 55]	18.7 (4.6) [*n* = 47]
Voice appraisal subscales	BAVQ-R: Benevolence (0–18)	3.3 (3.9) [*n* = 343]	1.7 (2.0) [*n* = 157]	3.2 (3.0) [*n* = 84]	1.9 (2.2) [*n* = 55]	10.7 (3.1) [*n* = 47]
Voice appraisal subscales	BAVQ-R: Engagement (0–24)	4.3 (4.4) [*n* = 342]	2.9 (3.1) [*n* = 157]	3.6 (2.9) [*n* = 83]	2.9 (3.1) [*n* = 55]	11.7 (4.1) [*n* = 47]

*^a^see Supplementary Material B for full measures information.*

### Exploring profiles’ association with demographic information and clinical presentation outcomes

The next step was to analyse whether (a) LPs identified were associated with demographics (age, gender, ethnicity) and (b) LPs were differentially associated with outcome variables which represented participants’ clinical presentations (severity of voices, PTSD symptoms, global distress and motivation and pleasure difficulties). See Supplementary Material I for full statistical results of these analyses, with summaries presented later.

#### a) Demographic factors associated with profiles

The descriptive statistics for demographics are shown in Table 3, where there is a higher proportion of male gender across all profiles. LP1 ‘Adverse voices and relational trauma’ has the highest proportions of female and other non-male genders as compared to other profiles. The multinomial logistic regressions considering the association of demographics and LPs showed a significant association between individuals being female and other non-male genders and an increased likelihood of belonging to LP1 ‘Adverse voices and relational trauma’ as compared to LP2 ‘Low malevolent and omnipotent voices’ (OR [95% CI] = 0.472 [0.247; 0.901]). For ethnicity, only individuals within the ‘Any other’ ethnicity category, which has the lowest percentage, as compared to ‘White’, were significantly more likely to belong to LP4 ‘High benevolent voices’ as compared to LP1 ‘Adverse voices and relational trauma’ (reference profile; OR [95% CI] = 8.78 [2.75; 28.03]).

**Table 3.** Distribution of demographic covariates across profiles and full sample

Demographic covariate variables	Full sample (*n* = 345)	LP1 ‘Adverse voices and relational trauma’ (44.5%, *n* = 157)	LP2 ‘Low malevolent and omnipotent voices’ (25.2%, *n* = 84)	LP3 ‘Adverse voices yet low relational trauma’ (16.5%, *n* = 57)	LP4 ‘High benevolent voices’ (13.8%, *n* = 47)
	Mean (SD)	Mean (SD)	Mean (SD)	Mean (SD)	Mean (SD)
Age	39.6 (13.4)	41.5 (13.4)	38.7 (13.2)	37.3 (12.9)	37.5 (13)
Gender^a^	*n* (%)	*n* (%)	*n* (%)	*n* (%)	*n* (%)
Female	129 (37.4%)	70 (44.6%)	25 (29.8%)	17 (29.8%)	17 (36.2%)
Male	212 (61.4%)	85 (54.1%)	58 (69%)	40 (70.2%)	29 (61.7%)
Other	4 (1.2%)	2 (1.3%)	1 (1.2%)	0 (0%)	1 (2.1%)
Ethnicity category^b^					
White	206 (59.7%)	101 (64.3%)	51 (60.7%)	34 (59.6%)	20 (42.6%)
Black or mixed Black	72 (20.9%)	29 (18.5%)	19 (22.6%)	12 (21.1%)	12 (25.5%)
South Asian or mixed South Asian	36 (10.4%)	19 (12.1%)	6 (7.1%)	8 (14%)	3 (6.4%)
Any other	31 (9%)	8 (5.1%)	8 (9.5%)	3 (5.3%)	12 (25.5%)

a Please note that when considering statistical testing of the demographic covariate associations, due to sample size (i.e., only 4 other non-male respondents), female and other non-male gender categories were collapsed together, thus the analysis includes a binary variable of ‘male’ and ‘female and other non-male genders’.

b Please see Table 2 in Supplementary Material B for full details of ethnicity for each of the categories in line with the larger AVATAR2 RCT.

#### b) Clinical presentation outcomes associated with profiles

Table 4 shows the distribution of clinical outcomes in the full sample and across LPs. The profile-specific intercept regressions showed that all profiles have a unique influence on the distal outcomes independent of the influence of the demographic covariates accounted for in the model (treated as binary: male/female and other non-male genders), and white/not-white (i.e., all other ethnicities)). When comparing these profile-specific intercepts in equivalence omnibus chi-square tests, these indicated that LPs were only significantly different in their associations with voice severity (*X*^2^(3) = 10.4, *p* = 0.015) and global distress outcomes (depression: *X*^2^(3) = 8.4, *p* = 0.038*; anxiety: *X*^2^(3) = 10.2, *p* = 0.017; stress: *X*^2^(3) = 10.3, *p* = 0.016), yet not with PTSD (*X*^2^(3) = 7.2, *p* = 0.066) and motivation and pleasure difficulties (*X*^2^(3) = 6.7, *p* = 0.083) outcomes, after controlling for covariates’ influences.

**Table 4.** Latent profiles and associated clinical outcome distal variables

		Full sample (*n* = 345)	LP1 ‘Adverse voices and relational trauma’ (44.5%, *n* = 157)	LP2 ‘Low malevolent and omnipotent voices’ (25.2%, *n* = 84)	LP3 ‘Adverse voices yet low relational trauma’ (16.5%, *n* = 57)	LP4 ‘High benevolent voices’ (13.8%, *n* = 47)
Distal variables	Measure^a^ (range)	Mean (SD)	Mean (SD)	Mean (SD)	Mean (SD)	Mean (SD)
Voice hearing severity	PSYRATS-H total subscale^b^ (0–44)	30.3 (4.5)	31.6 (4.0)	27.6 (4.9)	31.2 (3.9)	29.7 (3.7)
PTSD symptoms	ITQ PTSD dimensional score (0–24)	10.6 (7.4)	13.5 (6.9)	7.7 (6.2)	5.1 (6.2)	12.7 (6.4)
Global distress	DASS–21: Depression subscale (normal: 0–9, mild: 10–13, moderate: 14–20, severe: 21–27, extremely severe: 28–42)^c^	22.9 (11.3) Severe	27.4 (10.7) [*n* = 155]^4^ Severe	17.1 (10.1) Moderate	18.8 (10.7) [*n* = 55] Moderate	23.4 (9.6) Severe
Global distress	DASS–21: Anxiety subscale (normal: 0–7, mild: 8–9, moderate: 10–14, severe: 15–19, extremely severe: 20–42)^c^	19.5 (10.3) Extremely severe	23.4 (9.6) [*n* = 154] Extremely severe	13.6 (8.8) Moderate	15.3 (9.4) [*n* = 55] Severe	22.1 (9.6) Extremely severe
Global distress	DASS–21: Stress subscale (normal: 0–14, mild: 15–18, moderate: 19–25, severe: 26–33, extremely severe: 34–42)^c^	23.0 (10.2) Moderate	27.3 (9.3) [*n* = 154] Severe	17.1 (7.9) Mild	18.3 (10.8) [*n* = 55] Mild	24.9 (8.8) Moderate
Level of motivation and pleasure difficulties	CAINS: motivation-pleasure subscale (0–36)	15.8 (7.5)	16.9 (7.4)	13.2 (6.6)	16.4 (7.8)	16.3 (8.1)

a see further information about measures in Supplementary Material B.

b DASS-21 is scored via doubling summed scores for each subscale given it is the short form for the scale.

c Missing cases are represented here for the descriptive statistics however multiple imputation was utilized for missing values on distal outcomes for the statistical analyses.

Thus, considering the pairwise *z*-tests for the significant outcomes, LP1 ‘Adverse voices and relational trauma’ closely followed by LP3 ‘Adverse voices yet low relational trauma’ (not statistically different from each other) had the highest voice severity scores, relative to the full sample, which were both statistically different in their influence of such outcomes when compared to LP2 ‘Low malevolent and omnipotent voices’ and LP4 ‘High benevolent voices’ (not significantly different between themselves) which were associated with lower voice severity scores. Additionally, LP1 ‘Adverse voices and relational trauma’ was significantly associated with individuals having the worst emotional distress (ranges: severe depression and stress, extremely severe anxiety) as compared to better outcomes in LP2 ‘Low malevolent and omnipotent voices’ (ranges: moderate depression and anxiety, mild stress). Specifically for depression, LP1 ‘Adverse voices and relational trauma’ and LP4 ‘High benevolent voices’ (second highest mean score of severe depression) are significantly different in their association with depression outcomes. See Figure 5 in Supplementary Material I for a graphical representation of these results.

Table 5 provides a visual summary of all findings.

**Table 5.** Summary of Latent Profile results and clinical presentation outcome with traffic light system representing severity*

Profiles	Indicator variables	Demographics covariates	Outcome: Voice severity – PSYRATS-H	Outcome: PTSD symptoms – ITQ	Outcome: Global distress			Outcome: Level of motivation and pleasure difficulties – CAINS MAP subscale
					DASS-21 Depression	DASS-21 Anxiety	DASS-21 Stress	
	Full sample: high fearful attachment, trauma, trauma-voice association, malevolent, omnipotent voices, and voice resistance; low benevolent voices and voice engagement.		*Influence of profiles on the PSYRATS-H is significantly different.*	*Influence of profiles on PTSD is not significantly different. Descriptive information considered.*	*Influence of profiles on depression is significantly different.*	*Influence of profiles on anxiety is significantly different.*	*Influence of profiles on stress is significantly different.*	*Influence of profiles on the MAP subscale in the CAINS is not significantly different. Descriptive information considered.*
LP1 ‘Adverse voices and relational trauma’ (44.5%, *n* = 157)	This profile is higher than the full sample for fearful attachment and malevolent voices. Like the full sample this profile has high fearful attachment, trauma, trauma-voice relatedness, and high omnipotent voices which are highly resisted to. Like the full sample this profile has low benevolent voices and voice engagement.	More likely to be female and other non-male genders as compared to LP2. More likely to be ‘White’ than ‘Any other’ ethnicity when compared to LP4.	Differentially associated with having the most severe PSYRATS-H score as compared to all profiles except LP3.	Highest PTSD scores.	Differentially associated with having the worst depression score (severe), as compared to LP2 and LP4, not LP3.	Differentially associated with having the worst anxiety score (extremely severe) as compared to LP2 only.	Differentially associated with having the worst stress score (severe), as compared to LP2 only.	Highest score on difficulties with motivation and pleasure. Almost at cutoff score (17) indicative of negative symptoms.
LP2 ‘Low malevolent and omnipotent voices’ (25.2%, *n* = 84)	This profile is like the full sample level yet marginally lower on fearful attachment, trauma, trauma-voice relatedness. This profile is lower than the full sample on malevolent, omnipotent voices and voice resistance. It is very similar to the full sample with low benevolent voices and voice engagement.	More likely to be male as compared to LP1.	Differentially associated with having the lowest PSYRATS-H score as compared to all profiles except LP4.	Middle level of PTSD scores after LP1 and LP4.	Differentially associated with having the lowest depression score (moderate) as compared to LP1 only.	Belonging to this profile is differentially associated with having the lowest anxiety score (moderate) as compared to LP1 only.	Belonging to this profile is differentially associated with having the lowest stress score (mild) as compared to LP1 only.	Lowest score on difficulties with motivation and pleasure.
LP3 ‘Adverse voices yet low relational trauma’ (16.5%, *n* = 57)	This profile is substantially lower than the full sample on fearful attachment. It has a lower number of traumas and voice-trauma relatedness. Like the full sample it has high malevolent, omnipotent voices and very similar voice resistance, and low benevolent voices and voice engagement.		Belonging to this profile is differentially associated with having the 2^nd^ most severe PSYRATS-H score, as compared to all profiles except LP1.	Lowest PTSD score.	2^nd^ lowest depression score (moderate) yet belonging to this profile is not differentially associated with this outcome as compared to other profiles.	2^nd^ lowest anxiety score (severe) yet belonging to this profile is not differentially associated with this outcome as compared to other profiles.	2^nd^ lowest stress score (mild) yet belonging to this profile is not differentially associated with this outcome as compared to other profiles.	Just below highest score in LP1, in difficulties with motivation and pleasure, close to LP4. Score is close to cutoff score (17) indicative of negative symptoms.
LP4 ‘High benevolent voices’ (13.8%, *n* = 47)	This profile is like the full sample with high fearful attachment, trauma and trauma-voice relatedness and omnipotent voices. It is also similar yet slightly lower than full sample on malevolent voices and voice resistance. This profile is substantially higher than the full sample on benevolent voices and voice engagement.	More likely compared to LP1 to have ‘Any Other’ ethnicity instead of ‘White’.	Differentially associated with having the 2^nd^ lowest PSYRATS-H severity score as compared to all profiles except LP2.	2^nd^ highest PTSD score after LP1.	Differentially associated with having the 2^nd^ highest/worst scores for depression (severe) as compared to LP1 only.	2^nd^ worst anxiety score (extremely severe) yet belonging to this profile is not differentially associated with this outcome as compared to other profiles.	2^nd^ worst stress score (moderate) yet belonging to this profile is not differentially associated with this outcome as compared to other profiles.	Just below highest score in LP1, in difficulties with motivation and pleasure score, close to LP3. Score is close to cutoff score (17) indicative of negative symptoms.

*Note*: The color scheme represents a red-amber-green traffic light system to visually indicate severity of the clinical presentation outcomes across profiles (red: highest severity to green: lowest severity, with amber in between).

## Discussion

This study identified four statistically different subgroups of participants based on their experiences of fearful attachment, trauma, beliefs about trauma-voice relatedness, and voice appraisals: LP1 ‘Adverse voices and relational trauma’, LP2 ‘Low malevolent and omnipotent voices’, LP3 ‘Adverse voices yet low relational trauma’, and LP4 ‘High benevolent voices’. Differential profile membership was significantly associated with outcomes of voice severity and global distress but not for other clinical outcomes (internal experience of motivation and pleasure and related behaviors and PTSD). LP1 and LP2 reflect the two ends of the severity spectrum of adversities and voice relationships theorized in various models of voices, whereas the latter two revealed profiles (LP3, LP4) suggest a novel intersection of voice experiences and adversity. There were no clear patterns of demographic effects across profiles, with only gender association with LP1 and no differences across the more represented minority groups of Black and Asian backgrounds. The reflections from PPI consultations are incorporated throughout the discussion of results.

### Interpersonal adversity and impact on voices: support for existing models

In line with the existing models associating trauma and insecure attachment with voice appraisals and severity (e.g., Bailey et al., 2018; Berry et al., 2017; Scott, Rossell, Meyer, et al., 2020a), the larger LP1 ‘Adverse voices and relational trauma’ had higher interpersonal adversities linked to negative voice appraisals with worst voice severity and emotional distress outcomes. LP2 ‘Low malevolent and omnipotent voices’ had better scores on clinical outcomes alongside less adverse factors linked to substantially lower negative voice appraisals. PPI consultants discussed how the experience of cumulative trauma has a role in voice appraisals of powerlessness and persecution, in line with relational models which posit that interpersonal adversities can be internalized, individuals see themselves lower in power, mirrored in their voice relating (Hayward, 2003; Thomas et al., 2009) and more distressed responses to voices (Pilton et al., 2016). In this profile, trauma memories or dissociative trauma mechanisms, which can be predisposed by fearful attachment (Puckett et al., 2023), could result in trauma-related memories encoded in fragmented ways, re-experienced as negative voices (Hardy, 2017; Pilton et al., 2015).

The likelihood of belonging to the first more severe profile was associated with individuals being more likely to be female or other non-male genders as compared to the second profile. Increased severity and voice hearing distress has been reported in females in some studies (Murphy et al., 2010; Suessenbacher-Kessler et al., 2021; Toh et al., 2020), compared to males (see Barajas, Ochoa, Obiols, & Lalucat-Jo, 2015 for less favorable outcomes in males), with passive relating to voices explaining this association (Schlier et al., 2021) and male gender norms promoting the minimization of reporting voices (Goldstein & Lewine, 2000), powerlessness, or distress (Parent, Hammer, Bradstreet, Schwartz, & Jobe, 2018).

### Unexpected adversity and voice profiles

LP3 ‘Adverse voices yet low relational trauma’ and LP4 ‘High benevolent voices’ had an interesting combination of psychosocial factors, which may have been overlooked in the literature perhaps given previous studies’ different methodologies (e.g., isolated/paired psychosocial associations; Saunders et al., 2020). PPI consultants suggested that, where much lower fearful attachment and traumas in the LP3 group were reported, other ongoing environmental adversities (e.g., urbanicity, living in deprived areas) or difficult experiences (e.g., being from a minority group and experiencing subordination, discrimination, bullying), that were not captured in our demographic variables, can be impacting the individual. Where a continuing sense of threat results in hypervigilance which has been implicated in making negative information more salient shaping more negative voice-content (Larøi et al., 2019). As such, reflected in LP3 voices being highly malign and omnipotent, with the second-worst voice severity outcome.

Alternative non-trauma routes to voices in LP3 could be a genetic/family history (van Winkel et al., 2013), cognitive vulnerability, such as, strategies using punishment and worry for controlling unwanted thoughts, which are implicated with more psychological dysfunction and distress (Morrison & Wells, 2000), and low self-esteem (Williams et al., 2018). This group’s lower emotional distress could be linked with the lower fearful attachment suggesting more robust attachment styles (e.g., secure) may contribute to this group’s better emotion regulation and coping (e.g., lower emotional hyperactivity linked to positive psychosis symptom vulnerability; Berry et al., 2017). It may also be indicative of aspects not captured in this study, such as voice acceptance lowering negative distress from the lesser emotional resistance to voices, impacting voice distress (Varese et al., 2016) and psychological flexibility’s positive impact on general emotional wellbeing (Morris et al., 2014).

LP4 ‘High benevolent voices’ has a substantially higher level of benevolent voice appraisals, highly engaged with, alongside reported high fearful attachment, trauma, and omnipotent voices, similar to the first profile. With less supportive attachment relationships to provide a buffer to traumas reported, PPI consultants and previous literature suggest that other experiences from an upbringing in cultures and religious contexts, with helpful positive beliefs in relation to voices, could provide a soothing or accepting stance for voice hearing (Larøi et al., 2014). Voices then can be experienced as powerful, as seen in this group, yet this power is reported as more benevolent (e.g., Cottam et al., 2011). Of note, being of ‘Any other’ (and not other core minority Black and Asian backgrounds) rather than ‘White’ ethnicity was associated with this profile (as compared to LP1) and further information would be needed to interpret this finding.

In line with other studies showing a positive relationship between benevolent voices and lower voice distress (Sanjuan et al., 2004), the greater voice engagement may help lower the intensity and severity of voices in LP4 (Sayer et al., 2000), potentially due to a voice dialogue where the hearer is alongside the voice, rather than a victim, as suggested by PPIs. However, when considering LP4’s greater emotional distress, in line with psychological flexibility models, an appraisal of voices as omnipotent in this profile (regardless of it being a benevolent intention) can imply greater judgment toward these experiences and be responded to literally in subordinate ways which can impact emotional wellbeing (Gilbert et al., 2001). Additionally, experiencing voices as friendly in this profile, especially in the context of potentially impoverished social interactions, may be associated with retreating to the voice relationship for comfort/companionship (Miller et al., 1993), impacting social functioning and seeking treatment support (Favrod et al., 2004). Of importance, the current study’s participants are seeking treatment of a relational kind. Yet, the social withdrawal (LP4’s average motivation and pleasure difficulties being close to cutoff indicative of negative symptoms) may have associations with higher chronicity of symptoms, previously proposed to be that with more turbulent patients they are prone to more positive relationship with voices, and poorer outcomes including emotional distress (Favrod et al., 2004).

### Limitations

This is a cross-sectional study where LPA identifies subgroups dependent on the variables included which was limited to the available sample size (Tein et al., 2013; see Supplementary Material C). LPA is recognized as not sufficient to prove that profiles found will exist as tangible groups of people in other data samples, they provide guides for clinical consideration, but are not suggested as reified profiles (Williams & Kibowski, 2016). It thus should be noted that this study sample is specific individuals distressed by persistent voices who view a relational AVATAR treatment approach as relevant to them which can influence findings, since other voice hearers might not conceptualize their voice experience as a relationship (e.g., Chin et al., 2009). Thus, this is not representative of all voice hearers, some of whom might not engage with services.

The trauma measure (i.e., TALE) used, although a clinically useful measure, requires further better-quality evidence (Airey et al., 2023) which incurs limitations for the reliability of interpreting trauma associations in this study. The selection of the shorter (Mini-TALE) measure in the AVATAR2 trial assessment protocol (Garety et al., 2021, 2024) was intended to minimize participant burden from using longer intrusive questionnaires (Fornells-Ambrojo et al., 2017). This measure captures only the cumulative elements of trauma, without differentiating childhood and adulthood trauma. Given evidence about the specific role of childhood trauma (Stanton et al., 2020) and the cumulative or shared impact of adversity across different life stages (Pastore et al., 2020; Trauelsen et al., 2015) in psychosis, the findings of the current study require replication with a more robust trauma measure that distinguishes trauma across the lifespan and includes other trauma types, which PPI feedback for all LPs highlighted as important, such as, poverty, discrimination, and urbanicity factors (Fett et al., 2019; Varchmin et al., 2021). There are inconsistent findings related to associations of trauma type and hallucination modality (c.f. Barnes et al., 2023). Thus, due to statistical constraints related to model convergence (see Supplementary Materials C for further consideration), this study was not able to separate the types of interpersonal traumas (c.f. Begemann et al., 2022) since this would complicate the model and increase error where with the current sample size this would yield meaningless profiles. Non-trauma-related factors such as comorbid difficulties (e.g., anxiety and depression, autism spectrum disorder) could also have a mediating role for all profiles, perhaps impacting cognitive functioning and schemas of self-other, as such, consideration of such further factors are important and suggested by PPIs in future studies.

Interpretability of variables in a clinical utility sense were continually negotiated, however, for analysis ethnicity categories needed to be collapsed into mixed groups and binary codes making findings harder to interpret (i.e., association of ‘Any other’ ethnicity and increased likelihood of membership to LP4 ‘High benevolent voice’). This highlights the limitations in investigating the role of ethnicity within studies not specifically powered for this, as is the case of the current study.

### Clinical and research implications

The current findings highlight the importance of thorough assessments of adverse experiences, alongside careful formulations of the meaning traumas have in reference to voices and how they relate to ways the voice hearer does/does not and has/has not been able to form safe attachments with others. Given profiles from a sample of individuals with distressing voices have both negative and positive voices, asking patients for detailed descriptions and interpretations of their voice could aid clinicians to not miss information, such as, benevolent appraisals and engagement with voices valued by individuals and where fewer interpersonal adversities co-occur with distressing negative appraisals. Further exploration of other factors with stronger influences, including other adversities and comorbid difficulties not measured in this study, should be explored.

These case formulations could support clinicians and service users to discuss appropriate tailored treatment. For example, where relational therapies address past relationships with abusive caregivers via how these are represented in the voice interaction, aiming to change relating behavior with voices (O’Brien et al., 2021). Additionally, incorporating what the individual values about benevolent voices, especially respecting cultural or religious aspects, can help interventions to shape the voice relationship to how the individual wants, so they can feel safer and are meeting their own needs.

The findings in this study would benefit from further research considering generalizability of current profiles in other psychosis populations and non-clinical voice hearers, since this is a sample of individuals distressed by persistent voices who were willing to participate in the AVATAR2 RCT. A longitudinal investigation of identified profiles and their association with outcomes would be informative. Such as, AVATAR2 studies considering whether identified profiles respond differently to the AVATAR treatments received, which may inform understanding of how best to target treatments to different groups. The invaluable collaboration from PPIs in this study, in shaping variable selection and meaning making of results, is an important demonstration of how Experts by Experience can and should be incorporated in future research so that this can refine psychological support to meet the needs of voice hearers. Details of cultural background, historical wellbeing prior to voices, relationship to help, protective family experiences, occupational context, and developmental stages (stress/pressure) were raised by PPIs which should be incorporated in further research.

## Conclusion

This study uses LPA to provide new insights related to the complex interplay of interpersonal adversities co-occurring with positive and negative voice appraisals that are differentially associated with voice and emotional distress. Clinical information from this LPA can inform individualized assessments including careful consideration of voice appraisals, especially benevolent voices, and how these are linked to interpersonal adversities, to support decisions around helpful interventions. Information from the identified profiles could inform services, audits, and evaluations.

## Supporting information

Marotti et al. supplementary material Marotti et al. supplementary material

## Supplementary material

The supplementary material for this article can be found at http://doi.org/10.1017/S003329172500008X.
