# Supplementary material for: The role of trauma, attachment, and voice-hearer’s appraisals: a latent profile analysis in the AVATAR2 trial
Source: Psychol Med. 2025 Feb 27;55:e65. doi: 10.1017/S003329172500008X (PMC12080651; doi:10.1017/S003329172500008X)
Supplement: Marotti et al. supplementary material [file S003329172500008Xsup001.docx]

Supplementary materials to:

The role of trauma, attachment, and voice-hearer’s appraisals: a latent profile analysis in the AVATAR2 trial**.**

**Content:**

**Supplementary material A: Study participant flow** (Pages 2)

**Supplementary material B: Measures and participant characteristics** (Pages 3-12)

**Supplementary material C: Indicator variable selection information** (Page 13)

**Supplementary material D: Model selection information** (Pages 14-15)

**Supplementary material E: Post-LPA analyses detailed information** (Pages 16-20)

**Supplementary material F: Patient and public involvement process** (Pages 21-29)

**Supplementary Material G: Full sample information** (Pages 30-38)

**Supplementary Material H: Graphical representation of latent profiles and their indicator variables distribution** (Pages 39-40)

**Supplementary Material I: Further details of post-LPA analyses results for demographic covariates and distal clinical outcomes and their association with profiles** (Pages 41-55)

**References** (Page 55-64)

**Supplementary Material A: AVATAR2 participant flow and criteria**

The AVATAR2 randomised controlled trial exclusion and inclusion criteria applied to all participants included in the current study can be seen described in detail in the Supplementary Figure 1 (adapted from the AVATAR2 DMC report, 2023). This figure also describes the participant recruitment and data collection flow. Please see Garety and colleagues (2021) for further trial details.

**Supplementary Figure 1.** Diagram for the AVATAR2 trial inclusion and exclusion recruitment and data collection for participants at baseline, adapted from AVATAR2 DMC report, 2023.


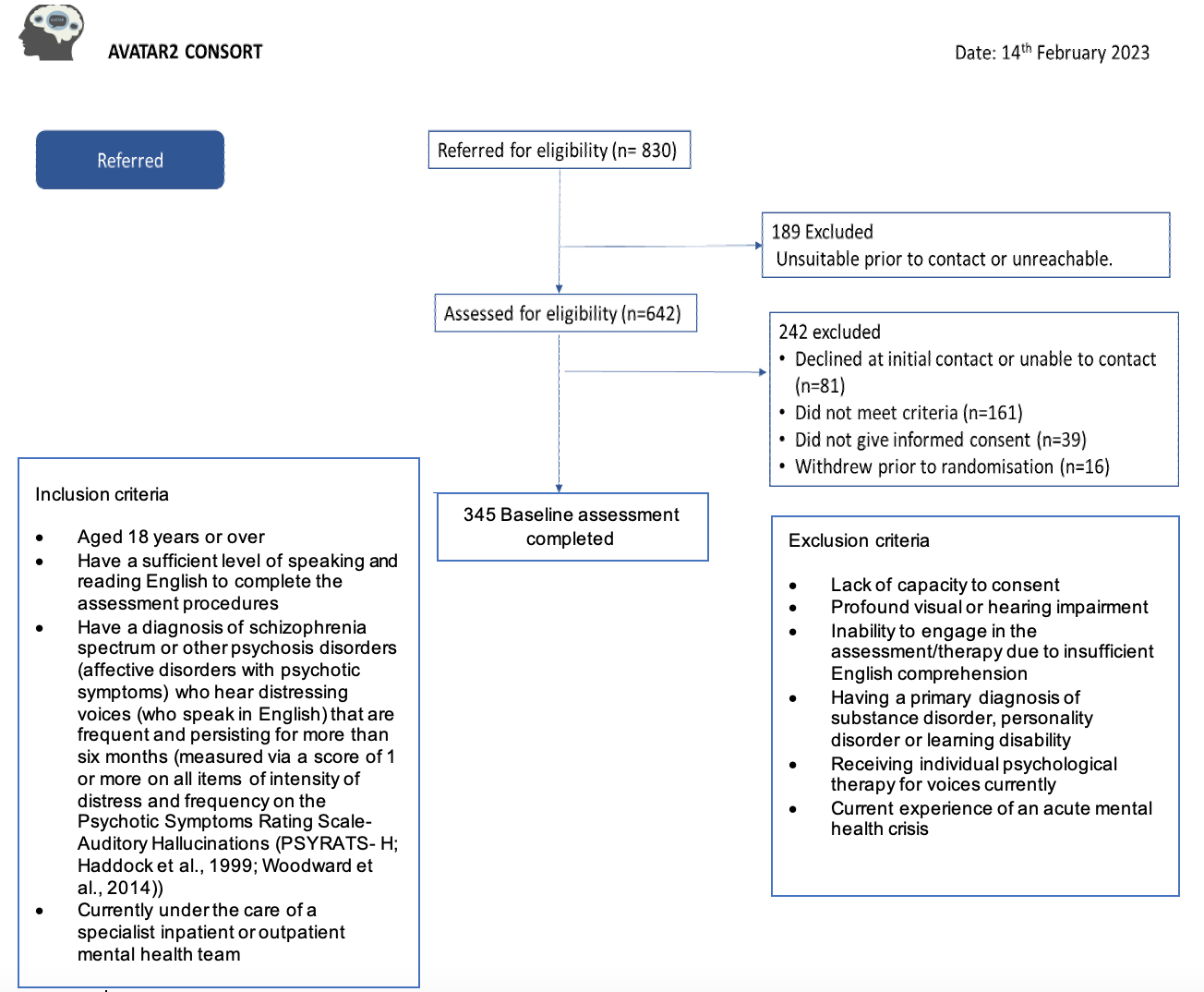


**Supplementary Material B: Measures and participant characteristics**

**Measures**

***Indicator variables***

**Attachment.** The Relationship Scale Questionnaire (RSQ; Griffin & Bartholomew, 1994), measuring on a seven-point Likert scale the extent to which participants agree with statements reflecting different adult attachment styles. In few studies this measure has been shown to have good validity and reliability, including across cultures (Wongpakaran, DeMaranville, & Wongpakaran, 2021; Schmitt et al., 2004). The RSQ full measure considers different adult attachment styles with an advantage of brevity. This study focused on the fearful attachment item alone, albeit with disadvantages of this constituting a single item score, it is in line with the project’s aim. The fearful attachment item score alone (i.e., “I am uncomfortable getting close to others. I want emotionally close relationships, but I find it difficult to trust others completely, or to depend on them. I worry that I will be hurt if I allow myself to become too close to others”) was focused on for direct consideration of this type of attachment. The range for the measure is 1-7 where scores of 7 indicate high fearful attachment.

**Trauma.** Trauma was assessed using a briefer measure adapted from the longer Trauma and Life Events (TALE) checklist, named Mini-TALE, based on a rationale of decreasing the questionnaire burden on participants (Fornells-Ambrojo et al., 2017). The TALE was recently designed as a psychosis-specific trauma measure useful for clinical practice, albeit currently it has overall low-quality evidence, with only one methodologically adequate study with a small sample size, requiring further investigation of its psychometric properties (Airey, Taylor, Vikram, & Berry, 2023).

Additionally, it is recognised that using shorter measure, the Mini-TALE, was a decision made in the AVATAR2 protocol development. This is related to recognition of the load of longer questionnaires on participants, with emerging qualitative research of people with psychosis preferring shorter batteries, especially for young people with poorer outcomes (Fornells-Ambrojo et al., 2017) and when trauma measures can be perceived as more intrusive this corroborates where negative perceptions about measures are found to be associated with higher levels of burden (Yan, Fricker, & Tsai, 2020).

The Mini-TALE evaluates whether participants report, on a binary yes/no response, experiencing across their lifespan any of the 4 common traumatic life events: physical (“someone being physically violent towards you at home or in public (e.g. fights, assaults, mugging)?”), emotional (“being insulted, put down or humiliated (e.g. by family, friends or strangers)?”) and sexual (“sexual contact that either at the time or looking back on it now was unwanted (e.g. touching, talking, looking, penetration”) abuse, and neglect (“feeling unsafe, unloved or as if no one would protect you?”). A total cumulative score of the number of trauma subtypes reported as having been experienced is yielded from this measure, thus scores of 4 indicate responding yes for experiencing all trauma subtypes. For those where trauma is identified in the Mini-TALE (1+ score), another brief novel measure, Trauma Voice Associations Questionnaire (TVAQ; Woods, Jones, Alderson-Day, Callard, & Fernyhough, 2015), with psychometric properties that require further research (Tolmeijer et al., 2021), was used to capture beliefs about how trauma experiences link to voices (e.g., “voices say similar/the same things that were said to you/about you during any events?”). This measure yields a cumulative score, with a range of 0-3, with higher scores indicating higher beliefs of voice content having links to trauma content.

**Voice appraisals.** The Beliefs About Voices Revised Questionnaire (BAVQ-R; Chadwick, Lees, & Birchwood, 2000) is a measure of participants’ beliefs about their voices (if more than one voice completed for dominant voice). Scores are summarised to five subscales, with higher scores reflecting greater occurrence of that subscale: extent of which voices are appraised as malevolent, benevolent, omnipotent, voice engagement and resistance. This measure is widely used in the literature and has acceptable psychometric properties, with high internal consistency for all subscales albeit test-retest reliability was not reported for this revised scale (Ratcliff, Farhall, & Shawyer, 2011). Construct validity is reported in several studies (e.g., Shawyer et al., 2003) and strong evidence for its convergent validity with other constructs (Andrew, Gray, & Snowden, 2008).

Higher scores in each subscale indicates higher occurrence of that voice belief, where scores are different for each subscale (i.e., voice malevolence: 0-18; voice omnipotence: 0-18; voice resistance: 0-27; voice benevolence: 0-18; voice engagement: 0-24).

***Distal outcome variables***

**Voice hearing severity.** The Psychotic Symptoms Rating Scale-Auditory Hallucinations (PSYRATS- H; Haddock, McCarron, Tarrier, & Faragher, 1999) is a semi-structure interview capturing hallucination characteristics yielding severity scores in domains of voice distress and frequency. This measure captures the severity of voices heard by participants, tapping into duration, location, and loudness of voices heard. It also considers the beliefs about the origin of the voices, the negative content they have and the degree of this voices’ controllability, as well as, the amount, intensity of distress experienced from hearing voices and how much they disrupt participants’ life. The range of the measure is 0-44 with higher scores indicating higher distress and frequency of voices thus higher severity of voices.

This scale is widely used with good psychometric properties established in psychosis literature, with strong inter-rater reliability, adequate test-retest reliability (Haddock et al., 1999), good internal consistency (Drake et al., 2007), with suggested modest convergent validity correlated against several measures (Ratcliff et al., 2011). A strength of this measure being that it is a broad multidimensional scale, with some debate about this complicating the interpretation as an outcome measure. Some argue that the total score should not be used over the separate dimensions (e.g., Steel et al., 2007). However due to a rationale of wanting to capture both frequency and distress of voices as indication of voice severity alongside managing the number of variables in this study a choice was made to use the total score to have the full outcome incorporated into this study.

**PTSD symptoms.** For those where one or more trauma(s) on the Mini-TALE was reported, The International Trauma Questionnaire (ITQ; Cloitre et al., 2018) was completed to explore PTSD-related problems. A total dimensional score is calculated across six PTSD items (scored on a five-point Likert scale), ranging from 0-24, where higher scores indicate a greater amount of PTSD symptoms. For participants where no trauma was reported they did not complete the ITQ. Such systematic missing PTSD data was recoded as 0 to specify individuals that did not complete this measure (i.e., reported not experiencing trauma) and to represent a lack of PTSD symptoms.

This measure shows satisfactory internal consistency across studies (e.g., Cloitre et al., 2019; Ho et al., 2020), with evidence of concurrent and discriminant validity (e.g., Hyland et al., 2017). There is factorial validity shown across different cultures also (Redican et al., 2021). The measure also shows a responsiveness to change which is indicative of a form of validity (Cloitre et al., 2021). Test-retest reliability shows fair to strong agreement albeit this is based on limited evidence (Ho et al., 2019).

**Global distress.** Emotional distress is captured in the four-point Likert scale responses on the Depression, Anxiety and Stress Scale (DASS-21; Henry & Crawford, 2005) yielding scores on subscales of depression, anxiety and stress. Albeit some discrepancies in the literature exist (Lee, Lee, & Moon, 2019), cut-off points are considered (i.e., depression: normal (0-9), mild (10-13), moderate (14-20), severe (21-27), extremely severe (28-42); anxiety: normal (0-7), mild (8-9), moderate (10-14), severe (15-19), extremely severe (20-42); and stress: normal (0-14), mild (15-18), moderate (19-25), severe (26-33), extremely severe (34-42)). Of note, total scores are computed by doubling the summed scores for each subscale given it is the short form for the scale.

This measure has a strength of being a single measure for depression, anxiety and stress together which has been well used as a clinical tool (Henry & Crawford, 2005; Ng et al., 2007). A review of the psychometric properties of the measure concluded that there was high quality internal consistency suggesting it is comprehensive in including key concepts, however, criterion validity is only high for the depression subscale (Lee et al., 2019). Low-quality of evidence for the reliability of this measure was noted and further research is warranted (Lee et al., 2019). It was also noted that having better established cut-off points for this measure would improve its applicability (Lee et al., 2019).

**Motivation and pleasure difficulties.** The Clinical Assessment Interview for Negative Symptoms (CAINS; Kring, Gur, Blanchard, Horan, & Reise, 2013) is a semi-structured interview with prompts to explore how participants spend their time and who with. To address existing measures’ limitations, the CAINS was developed (Horan et al., 2006) where scales are noted to relate to real-world functioning (Kring et al., 2013). This measure is found to have good internal consistency, test-retest reliability, inter-rater agreement, and strong convergent and discriminant validity (Horan et al., 2011; Kring et al., 2013).

This study focuses on the 9-item Motivation and Pleasure (MAP) subscale which is a measure of individuals’ motivation and pleasure difficulties (or negative symptoms) across recreational, vocational and social domains. Participants’ responses are scored on a five-point scale with higher scores indicating greater impairment on their motivation and pleasure across domains (‘no impairment’ to ‘severe deficit’). The range for scores are 0-36. Suggested cut-off score for identifying persistent negative symptoms is 17 for the MAP subscale as such this is loosely used to interpret findings (Li et al., 2018).

***Distal covariate demographic variables***

Participants’ age, gender and ethnicity were considered in their association with LPA profiles. There had to be careful consideration about representing groups of ethnicities in a meaningful way to account for this influence in a way that has clinical utility and real-life consideration. In accordance with the larger AVATAR2 RCT, the chosen ethnicity categorisation (see Supplementary Table 1 for full information of ethnicity classification categories) for this study aims to minimise the inclusion of low-powered small specific groups (i.e., not having sufficient numbers to capture the nuance in single ethnicity experiences) and includes groups that are meaningfully acknowledging potential shared experiences of racial discrimination (e.g., one group with Black African, Caribbean, British or mixed Black ethnicity) based on research about ethnicity experiences in psychosis including single and multiple ethnicities (e.g., Edge et al., 2016; Degnan, Berry, Crossley, & Edge, 2023; Devonport et al, 2022). it is acknowledged that the chosen ethnicity categories were not in line with United Kingdom government standards for ethnicity data (Office for National Statistics, 2022) and it still includes heterogeneity and assumptions of shared experiences, especially in the mixed ‘Any other’ group, which might not allow for interpreting any meaningful experiences, for example, of shared discrimination. However, in line with qualitative studies highlighting similarities in ethnicity experiences, such as, shared inequalities, experience of Blackness, which had inclusion criteria often encompassing people with single or multiple ethnicities (e.g., Degnan et al., 2023; Devonport et al, 2022; Islam, Rabiee, & Singh, 2015; Lenoir & Wong, 2023), alongside aims to not have extremely small groups of single ethnicities which underpower any analysis including these variables, the categorisation chosen was the compromise struck.

Due to low numbers in categories of gender and ethnicity, resulting in under-powered tests when controlling for such variables, a decision of having to use binary codes (e.g., white/not-white ethnicities (i.e., ‘Black & mixed Black’, ‘South Asian & mixed South Asian’ and ‘Any Other’) and Male/Not-male (i.e., Female and Other genders)) was made. This loses substantial detail, impacts the ability to interpret such findings and potential clinical utility for such results. However, it was a necessary step to be able to control for such variables in the analysis. This was a difficult decision given Patient and Public Involvement (PPI) consultations for this project highlighted the importance of culture and ethnicity on experiences of voices. This could not be incorporated in the current project and highlights areas for future research.

**Supplementary Table 1.** *Table of the detailed ethnicity classification categories.*

| Ethnicity subtype for analysis | Frequency  n=345 (%) | Includes (n) | Details as on the database (n) |
| --- | --- | --- | --- |
| White | 206 (59.7%) | White (203) | White (203)  White British Italian (1)  White Scottish (2) |
| Black or mixed Black | 72 (20.9) | Black African (29)  Black Caribbean (22)  Black Other (10)  Black Mixed Ethnicity (11) | Black African (29)  Black Caribbean (22)  Black-other (6)  African American (1)  Black-British(2)  Black (1)  Black-white (1)  British black and white African (1)  Mixed white and black African (3)  White British-black Caribbean (quarter caste)/mixed white British & black Caribbean (2)  Mixed white Irish & black Ghanaian (1)  Mixed/mixed race white and black Caribbean (2)  Mixed race (Afro-Caribbean and white) (1) |
| South Asian or Mixed South Asian | 36 (10.4%) | Pakistani (16)  Indian (11)  Bangladesh (3)  Bengali (2)  South Asian Mixed Ethnicity (4) | Bangladesh British/British Bangladeshi (2)  British Bengali/Bengali (2)  Asian-Mauritian (1)  Bangladeshi (1)  Pakistani (15)  Indian (10)  British Pakistani (1)  Indian Scottish (1)  Mixed South Asian – white (1)  Mixed white & Pakistani (1)  Mixed-Sri Lankan (1) |
| Any Other | 31 (9.0%) | East Asian (6)  Other (12)  Unclear from description (e.g., British) (3)  Mixed ethnicity, excluding people included Black or South Asian (9) | Vietnamese Chinese (1)  Chinese (2)  Vietnamese-British (1)  British-  Asian (1)  Scottish Asian (1)  British (2)  Latino (1)  Turkish (1)  Greek (1)  Mauritian (1)  Albanian (1)  Middle eastern (1)  Arab-Moroccan (1)  Arabic (1)  Afghan (2)  Arabic Algerian (1)  Kurdish (1)  Scottish (1)  Mixed-other (1)  British-Columbian (1)  Mixed ethnic background (2)  Mixed race (1)  Turkish & Egyptian (1)  African-British (1)  Turkish-Cypriot (1)  White-Asian (1)  Mixed white-Japanese (1) |

**Supplementary Material C: Indicator variable selection information**

Consideration was given to the total number of indicator variables included in the analysis to reduce model convergence issues of having too many indicator variables for the available sample size (Dalmaijer, Nord, & Astle, 2022). This is in line with limited literature considering statistical power in LPA methodology (see Tein, Coxe, & Cham, 2013). Indicator variables selection had to be balanced with ensuring that indicator variables remained recognisable and meaningful to clinicians so identified profiles have potential clinical utility. For example, not collapsing subscales of voice appraisals since each indicates to a different characteristic of voices (e.g., malevolent, omnipotence). Thus, in consultation with Patient and Public Involvement (see Supplementary Supplementary Material F) co-produced choices of indicator variables and certain measures retained were made (i.e., Beliefs and Voices Revised Questionnaire (BAVQ-R; Chadwick, Lees, & Birchwood, 2000) chosen for voice appraisals instead of alternative measures (i.e., Voices Acceptance and Action Scale (Shawyer et al., 2007), or Voice Power Differential Scale (Birchwood et al., 2000)), and keeping the Trauma-voice association questionnaire (TVAQ; Woods et al., 2015)).

**Supplementary Material D: Model selection information and model comparison statistics**

The optimum profile solution for the data is determined by comparing model fit statistics of successive estimated models with incrementally higher number of profiles (starting with a two-profile solution). The Vuong-Lo-Medell-Rubin Likelihood Ratio test (VLMR-LRT; 3 Mendell, & Rubin, 2001), and Bootstrap Likelihood Ratio Difference test (B-LRT; Nylund, Asparouhov, & Muthén, 2007) alongside Akaike Information Criterion (AIC), Bayesian Information Criterion (BIC) and entropy-based criterion values (Asparouhov & Muthén, 2012; Geiser, 2013) are successively compared to inform model fit. These are presented in Supplementary Table 2.

The VLMR-LRT and B-LRT compare the K model, which is the current model with K number of profiles, against the K-1 model, which is the model with one less profile estimated, with B-LRT confirming against the K-1 model using a bootstrap procedure (Asparouhov & Muthén, 2012; Geiser, 2013). A significant p value (p<0.05) indicates that the K model is likely to fit the data better than the K-1 model. While a non-significant finding indicates that the model with one less profile is a better fit, suggesting that the more parsimonious model is preferred. Results showed significant B-LRT at successive models indicating using a more reliable bootstrap procedure that the four-profile is better than the three-profile however it also indicates that a five-profile is better than a four-profile model. When the AIC and BIC values lower between models, as seen in the results below, they indicate that the model fit with more profiles is better, while accounting for model parsimony where each additional profile increases the number of model parameters to be estimated (Geiser, 2013). A higher entropy value (closer to 1) also indicates a higher accuracy of the classification thus whether individuals can be reliably allocated to the identified profiles in the model (Masyn, 2013; Nylund et al., 2007). A non-significant VLMR-LRT p-value and a higher entropy value at the five-profile solution is indicating a higher classification accuracy for the four-profile model solution. Taken together, the best fitting model was suggested at the four-profile solution.

In considering interpretability of the chosen profile solution the size of the mean profile assignment probabilities (≥ 0.8) can be considered in whether there is a greater than fifty-fifty probability of individuals belonging to the profile meaning it is less clear to interpret the profile responses (Geiser, 2013; Rost, 2003). The profiles’ mean class assignment probabilities showed high values (>0.8; LP1=.924, LP2=.916, LP3=.866, LP4=.945) indicating that individuals are assigned to their most likely LP with a high satisfactory precision and reliability.

**Supplementary Table 2.** Table with model comparison statistics for the different profile solutions.

| Model | AIC | BIC | Adj-BIC | VLMR-LRT (p=) | B-LRT (p=) | Entropy |
| --- | --- | --- | --- | --- | --- | --- |
| Two profiles | 12974.254 | 13070.343 | 12991.036 | 0.005 | <0.05 | 0.90 |
| Three profiles | 12773.283 | 12903.964 | 12796.106 | 0.013 | <0.05 | 0.82 |
| **Four profiles** | **12686.168** | **12851.44** | **12715.032** | **0.008** | **<0.05** | **0.84** |
| Five Profiles | 12626.258 | 12826.122 | 12661.164 | 0.198 | <0.05 | 0.83 |
| Six profiles | 12568.19 | 12802.646 | 12609.138 | 0.198 | <0.05 | 0.87 |
| *Note.* AIC=Akaike Information Criterion; BIC=Bayesian Information Criterion; Adj-BIC=Sample size adjusted Bayesian Information Criterion; VLMR-LRT=Vuong-Lo-Medell-Rubin Likelihood Ratio test; B-LRT =Bootstrap Likelihood Ratio Difference test. | | | | | | |

**Supplementary Material E: Post-LPA analyses detailed information**

Given the probability-based modelling procedure of LPA, there is a recognised error related to the uncertainty with how individuals are allocated to the profile they have the greatest probability of membership. The classification of individuals into profiles cannot be assumed to have the perfect number of members allocated to it (Bakk & Kuha, 2020; Bakk, Tekle, & Vermunt, 2013). A solution to this problem is an active area of ongoing research, where bias-adjusted three-step methods have been developed to be conducted within Mplus (Muthén & Muthén, 2017) which accounts for the accuracy/error in the profile allocation (Clark & Muthén, 2009; Vermunt, 2010; Bray, Lanza, & Tan, 2015) and were used in a series of post-LPA analyses: a) demographic variables: age, gender, ethnicity (i.e., the bias-adjusted R3STEP method), and b) clinical presentation outcomes, considered “distal outcomes” in LPA (i.e., the Bolck-Croon-Hagenaars (BCH) procedure; Bolck, Croon, & Hagenaars, 2004; Vermunt, 2010).

1. **Association of demographic factors with profile membership.**

To explore whether demographic variables (age, gender and ethnicity), considered “covariates” in LPA, had an association with profile membership (see Supplementary Figure 2 for the visual representation of this model) multinomial logistic regression analyses were conducted in Mplus using the bias-adjusted R3STEP method (Asparouhov & Muthén, 2014). For these analyses, two profiles in turn were compared as the independent variable allowing a comparison of the odds ratio of each demographic covariate’s association with the likelihood of a participant being a member of one profile relative to a reference profile, chosen based on its sample size.

**Supplementary Figure 2.** Visual representation of the covariate effect of age, gender and ethnicity on the Latent Profile (P), where the arrows represent that P is being regressed on the covariates.


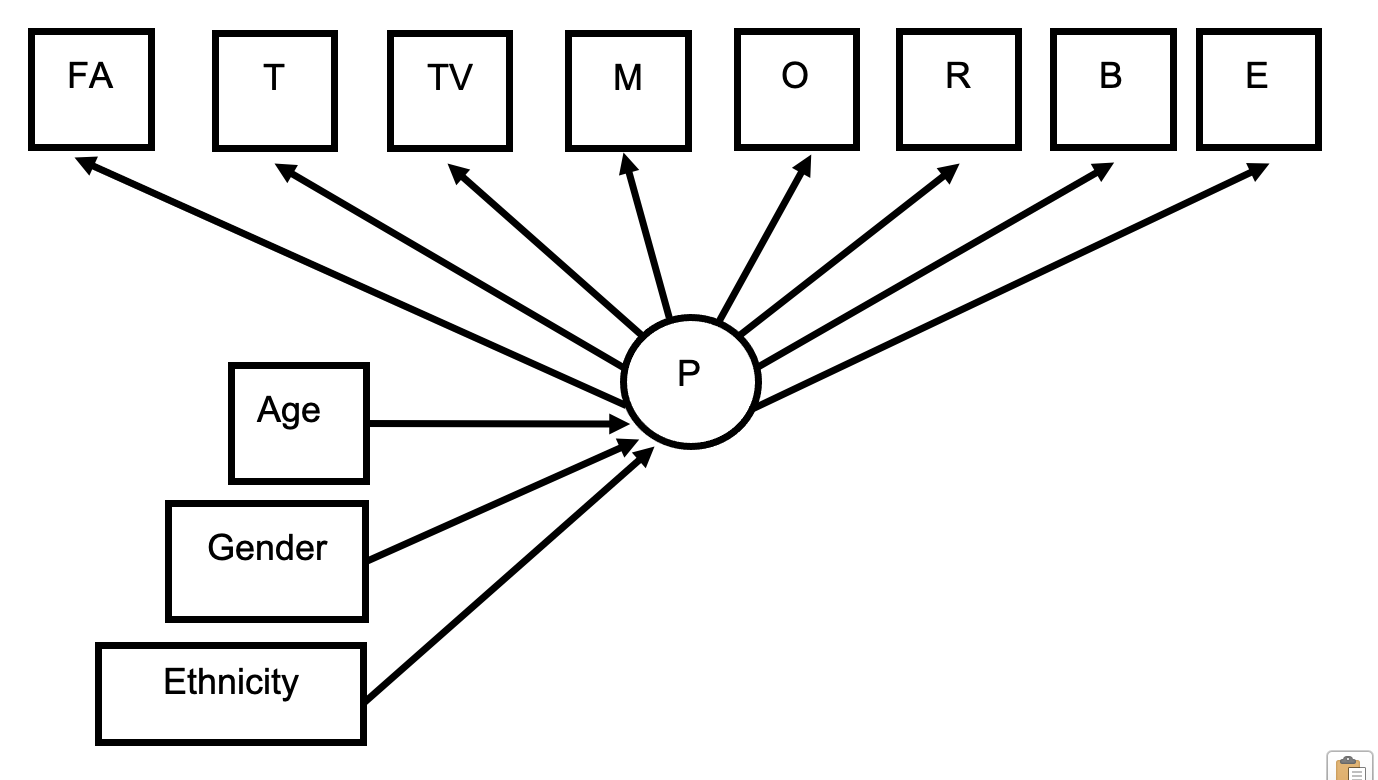


1. **Comparing clinical presentation differences across profiles.**

Comparing how profiles differ with respect to the distal outcomes of clinical presentations, of voice severity, PTSD symptoms, level of global distress and difficulties with motivation and pleasure (see Supplementary Figure 3 for the visual representation of this model), Since the model entropy was good (above 0.8), the recommended analysis conducted in Mplus was the Bolck-Croon-Hagenaars (BCH; Bolck et al., 2004; Vermunt, 2010; Asparouhov & Muthén, 2021), which can be used regardless of whether distal outcomes have equal or unequal variances across profiles (Bakk & Vermunt, 2016; Bakk et al., 2013, 2016; Bakk & Kuha, 2020).

The BCH method avoids profile changes by using inverse logits of computed classification errors for individuals as weights (BCH weights), capturing the measurement error of the latent profile variables (Bolck et al., 2004; Vermunt, 2010). These weights are used in the second step of estimating the general multinomial logistic regression model considering distal outcomes conditional on latent profile variables (captured within the BCH weights) while controlling for covariates (McLarnon & O’Neill, 2018). That is, while exploring LPs’ association with distal outcomes, it was important to control for demographic covariate effects to account for correlations between covariates and distal outcomes as previous studies suggest (e.g., Larøi et al., 2014). First, using multiple imputation for missing data introduced further steps to the BCH coding; an unimputed LPA was ran to save BCH weights then using this version of the data an imputed dataset was created for the regression of distal outcomes (section 11.1 of Asparouhov & Muthén, 2021). Second, based on recent papers and Mplus guidance (e.g., Nylund-Gibson, Grimm, & Masyn, 2019; McLarnon & O’Neill, 2018; Garber, 2021; Morin et al., 2020; Bakk & Vermunt, 2016; Mplus Discussion forums), a more advanced manual three-step BCH procedure was required where this was an extension of estimating effects which has been rarely used with LPA (McLarnon & O’Neill, 2018). From the literature and forums, it was understood that within the available coding on MPlus it is not possible to directly regress latent profiles on the outcome variables when controlling for covariates. The steps required for accounting for this is regressing the demographics on the identified latent profiles (as defined by the indicator variables captured in a weighted measure in the BCH procedure) then latent profiles are technically conceptualised as dummy covariates as in a linear regression where it then changes the intercept within the estimated model with outcomes and demographic covariates accounted for.

Therefore, when interpreting this complex analysis, the profile-specific regression intercept outcomes represent the influence of each LP on the mean of a distal outcome beyond the influence of covariates and other profiles (based on guidance from Mplus Discussion forum, 2014). To see whether these profile-specific intercepts are significantly different across profiles while controlling for covariates, omnibus Wald chi-square tests are computed for each outcome. For the outcomes that the omnibus chi-square tests are significant, then pairwise z-test comparisons (using the delta method; Raykov & Marcoulides, 2004) of the profile-specific intercepts, indicating how profile membership is associated with the outcome after accounting for covariate effects, will be considered for each profile’s separate association with that outcome.

**Supplementary Figure 3**. Visual representation of outcome variables of severity of voices (V), PTSD symptoms (PTSD), global distress subscales (D), and level of motivation and pleasure difficulties (MAP) being regressed on the latent profile model (P), indicating P’s influence on distal outcomes. Covariates effects of age, gender and ethnicity are depicted by the presence of covariates in boxes without arrows since its effect on the whole model is accounted/controlled for.


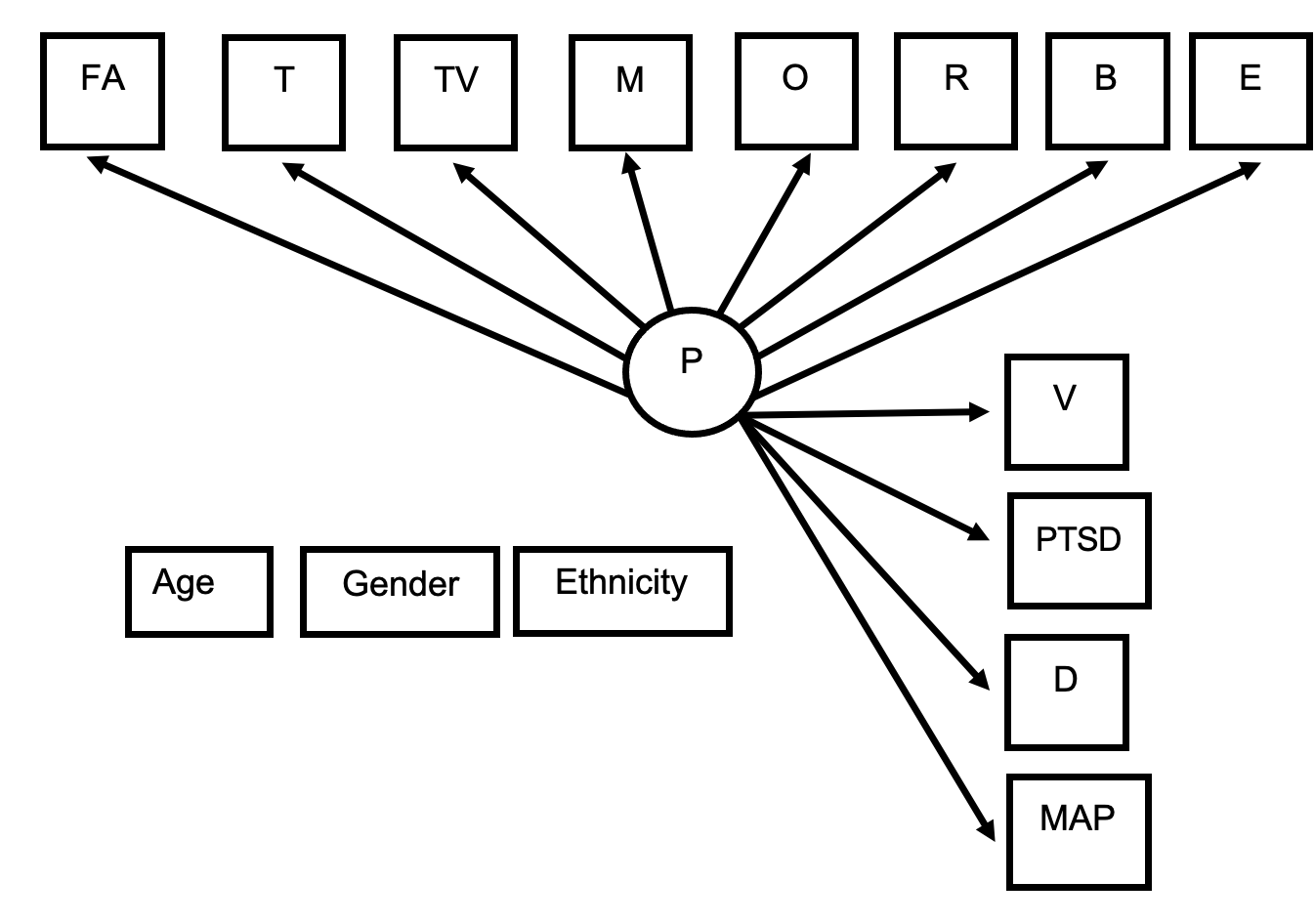


It is important to note that when a model does not control for covariates the equivalence tests are not the same, instead, that separate test is considering the equivalence of outcome means across profiles (to consider how profiles differentially predict these outcomes). Since they are different analyses, one cannot provide a direct comparison for this model of when covariates are controlled for and when not, as such not represented in this study.

**Supplementary Material F: Patient and public involvement process**

This study considered meaningful involvement of people with lived experiences at a) study design one-to-one consultations, for considering indicator variables selection, importance and measures to be retained, and b) post-analysis one-to-one consultations, for discussing and interpreting the LPA profile results. Consideration about how to present complex information in an accessible way was considered throughout consultations to ensure PPI feedback could be as rich and meaningful as possible. The trial provides structures of support for PPIs (i.e., training, termly group meetings).

#### a) Patient and Public Involvement pre-analysis stage – Indicator variable selection consultation.

The PPI feedback especially supported the decision-making process of prioritising measures included in the LPA. PPIs felt that trauma and voice appraisal variables were important to retain as these were essential for understanding voices and for treatment. The attachment variable was retained also since some PPIs felt it can capture traumatic relationships with significant caregivers which can have links to negative voices experiences. Given that PPIs consistently stated the importance of considering how voice relationship is related to trauma, the Trauma Voice Associations Questionnaire was retained (TVAQ; Woods et al., 2015). PPI feedback was also sought for the selection of the voice appraisals measure being the BAVQ-R (and not the Voices Acceptance and Action Scale (Shawyer et al., 2007), or Voice Power Differential Scale (Birchwood et al., 2000)) since most PPIs felt this was a more detailed and comprehensive measure. See Supplementary Table 3 for detailed summary of feedback.

**Supplementary Table 3**. Table with summary of feedback and impact this made to the study (e.g., changes).

| Main learning points | Impact this made on the study | Not able to incorporate – noted in limitations section |
| --- | --- | --- |
| Family background is important to capture in studies about psychosis. This was including trauma and negative experiences especially, since these were referenced as potentially increasing later vulnerability to stress and developing voices. Many noted on trauma experiences being linked to voice content.  Early experiences were noted as essential to be considered in relation to trauma and then trauma impacting voices. However, attachment was noted by some as less important since there could be supportive and positive early relationships for people and trauma is capturing both when this is not happening and when trauma is related to non-primary caregivers.  All this was discussed as important to consider in reference to voice content and appraisal - allowing exploration of what voices are saying helping to link back to life and relational experiences, providing insight into the meaning of voices, the kind of relationship to voices – the power it has over you, beliefs of what they are. | These discussions provided support that we are including indicator variables in the LPA focusing on trauma, trauma-voice association, attachment and voice appraisals. Attachment was considered mostly in relation to trauma so consideration of this post-result will be discussed.  Thus, retaining trauma-voice association measure as one of the profile indicators was informed by these consultations since references to trauma and voice appraisals by PPIs made reference to considering content of voice in relation to trauma. Such a measure would capture this information.  PTSD impact could consider adverse experiences of cultural stigma and impact from trauma thus retained as a measure of distal outcome. | Not collected information about protective family experiences. |
| The majority vote was a preference for the longer measure called Beliefs about voices revised questionnaire. People shared this was chosen because it allowed tapping into trauma experiences questions and beliefs that explore control and power dynamics, it then covered what other measures (VAAS, VPDS) did and more. It is more detailed and has factors relevant to PPIs experiences that allows variety between people to be captured. | This project accordingly chose to retain BAVQ-R as the appraisal of voices measure out of the 3 considered. |  |
| Importance of considering cultural, gender, religious, ethnicity background and how this may impact on how people talk about their feelings and voice experiences (e.g., under -disclose). Including consideration of judgement from services in reference to cultural background (e.g. do clinicians expect emotion to be shown in a way that isn’t being shown (should be crying if sad) and judge this as not normal due to the lack of knowledge (assume person is not distressed, misunderstand)).  Thoughts shared about if a person’s cultural background may impact on whether there is stigma in their community about talking about mental health, whether cultural background responds with supporting person to ask for help or expectation to manage alone, then there could be fears of letting others down, others saying things that are not helpful. | Demographics used in the project will be focusing on ethnicity, gender both in how they impact the profiles and controlled for. | Detail about the cultural background and specific impact this has on the person both in their relating to their own difficulties and how services may see/judge them was not considered in this study. Stigma is not captured in this study also. |
| Relationship to the world including experiences of school and work, what stage person was in their development when voices occurred and impact this had in their stage of development was considered as important. Stress/pressure points related to different stages of life, transition points. | This project is considering demographic information about the onset age of voices and work/education status in the sample.  Including measure of motivation and pleasure difficulties as an outcome to capture some impacts of voices on social context. | Missing detail on social occupational context, impact this has on the person – stress/pressures related to these contexts not considered. |
| Timeline of difficulties overall including the context around it and other mental health difficulties (depression, anxiety, PTSD, social anxiety), considering what was it like before voices, giving insight into functioning and life before voices started. | This project is considering the onset age of voices as a demographic. It is also including clinical presentation outcome measures of emotional distress (anxiety, stress, depression), PTSD and motivation and pleasure difficulties across social, personal and vocation contexts. | Not able to include information of context and mental health difficulties prior to voice onset. |
| Importance of background of interventions and relationship to help previously – including path someone took to come to treatment may include a long journey to be diagnosed. This included comments around how there can be a fear around expressing voice experiences and consequences this may have (e.g. being sectioned, judgment from others). Also exploring fears person has around disclosing about voices in reference to impacting on the therapist, experiences in the past that were bad (e.g. being undermined/dismissed and stereotyped expectations) and this impacts on how person is in new support spaces, understanding what was unhelpful and how this differs. | Included number of years in contact with mental health services as a demographic in the sample. | Not able to capture details of relationship to help experiences, what allows someone to engage in the therapy based on previous experiences and the nuanced aspects commented on by PPIs |
| PPIs raised importance of considering times individuals are less connected to others – especially during developmental stages where this was key (e.g. child, teenager years) and this having a greater impact (e.g., bullied experiences, alienation – stigma, how voices may impact on ability to be present to relate socially). | This project considers social engagement by considering difficulties with motivation and pleasure (MAP subscale in the CAINS) as a way of capturing the aspects of relationship to others where higher scores may indicate to those less engaged/more isolated, having greater impairments. | Stigma/bullying/isolation aspects and the developmental stage details of this, impact and how this relates to voices are not properly captured in this study. |

***b) Patient and Public Involvement Post-analysis consultations – Interpreting the LPA profiles results****.*

Consultations completed with four PPIs explored PPI’s understanding of the identified LPA groupings and the distribution of the indicator variables within each profile. To present this complex model in an accessible way to PPI consultants, visual representations were utilised to support such discussions. A detailed summary of the feedback from these consultations can be seen below in Supplementary Table 4 with all identifiable information removed. These reflections are incorporated throughout the discussion of the results to ensure real-world insights are highlighted.

**Supplementary Table 4.** *Summary of the feedback from the 4 one-to-one consultations at the post-analysis stage, with all identifiable information removed.*

| General or Latent profile specific reflection | Feedback discussed |
| --- | --- |
| General | • Trauma and attachment distributions were commented on being consistent throughout, i.e., some relationship between high/low trauma and high/low fearful attachment?  • Considered that traumas can be very individual in what they mean to individuals  • Including considerations raised that traumas can be reported in relation to experiences from delusions and voice content  • Important to consider what constitutes for one person something might not be for another and needs to be considered  • Could there be a bias of whether individuals report less about benevolent voices?  • Demographic factors are important to consider across the groups to see if this impacts any experiences, including whether anything else the person experiences is part of these other characteristics (e.g., ASD, Anxiety)  • There was a lack of consensus from PPIs on profile names given above. Some felt that the general sense of the names were good others did not. One PPI considered about focusing on the differences between groups and naming that to support with simplicity of the message being put across.  • Considering the audience and if to be accessible then using more accessible words should be considered |
| LP1 ‘Adverse voices and relational trauma’ | • A few PPIs commented on feeling their experiences belonged to this group and this felt more familiar to them  • This was a group that was the most expected group in terms of experiences of trauma and attachment being fearful and the association with difficult voices (malevolent, omnipotent)  • Consideration was given that omnipotent voices could be associated with the trauma experienced e.g., omnipotent abusers impacting beliefs you develop as a child and this shapes experience of voices being omnipotent.  • Comments were made about the connection to what was witnessed during trauma and attachment experiences with what is experienced from hearing the voices. Consideration that trauma is expected to be the “fuel” behind negative voices being worse.  • Attachment and voice appraisals were considered briefly, e.g., it could be what increases experiences of voices being negative? Fearful attachment could project such experiences onto the voices (e.g., as a child the person is more vulnerable and cannot escape the trauma and this can mean they feel less powerful themselves and then they relate to voices as if the voices are all powerful and omnipotent.)  • Higher resistance to voices in this group was commented on as whether it can impact the intensity of the voices, that is, fuelling voices to be louder and worse towards the person. One PPI considered that voice resistance may be harder if the attachment experiences shape voice relationship given it is hard to resist against your significant caregiver. Thus fearful attachment experiences can provide a background to the relationship to resisting voices |
| LP2 ‘Low malevolent and omnipotent voices’ | • This was another group commented on as being expected as a group characteristic for voice hearing individuals (e.g., because if scared of a trauma then it would follow that you become scared of the voice but without scary experiences, with trauma being lower, then connects with voice being less “scary” have less scary voices).  • Trauma voice relatedness measure was higher than trauma numbers was commented by one PPI. Type of trauma could be important to consider as voice may be considering a specific trauma and being “efficient” at targeting this. |
| LP3 ‘Adverse voices yet low relational trauma’ | • Reasons why trauma being lower in this group while having difficult voices persist was considered:   1. It can depend on the type of trauma and the person when considering the impact of trauma, including that one trauma/stressor can be sufficient to considerably impact someone. 2. Perhaps there could be one experience over a long period of time that means that trauma is more impactful. 3. Trauma types we are not capturing in the study are being missed out - including witnessing others which might not be captured in the measures? 4. Day to day anxieties and experiences of ethnicity groups could be reflected in this group?   • Consideration that voices may be targeting areas that are of particular vulnerability for individuals (e.g., being from a minority group and being “punished for this”).  • Discussed about how voices can present themselves as effective at using difficulties and hard experiences of any kind to impact the individual as such voices can still be negative if less traumas present. Voices can be tapping into one impactful trauma.  • Could there be overwhelming experiences due to other needs that are not captured in these factors then leading to adverse voices?  • Could there be some consideration of this being a group that has a higher spectrum of symptoms, a genetic or familial aspect to symptoms. |
| LP4 ‘High benevolent voices’ | • PPIs considered that the more you engage with the voice the closer to a conversation it becomes, less abusive it feels and engaging and thinking with the voice. The voice can continue to repeat itself, but you feel more able to keep being able to develop from it and create a conversation (even challenge it). Closer to a dialogue which can help the person have more power and take part with being alongside the voice.  • Consideration that perhaps there may have been therapy and support, and protective experiences, previously, that has helped the voices be more benevolent in how they are experienced. For example:   1. positive and caring caregivers can support the existence of having some good voices experienced was considered. 2. Trauma in the context of a supportive family can be different in how impacts the individual. 3. Religion and the helpful beliefs this could bring in relation to voices being positive. Voice being soothing in your trauma given that faith is supporting you. 4. Upbringing in culture where voices are more accepted/not strange, less stigma then? (e.g., saints) If then hold belief voices are ok they could be more experienced as “nice voices”.   • Individual characteristics of this group explored which was suggested can support the more positive experience (e.g., self-worth and confidence built from other positive experiences in person’s life, person can then tap into this self-belief and helps you manage and deal with the voice, meaning voice feels more positive).  • Discussion about how trauma can be experienced and reported as trauma but it might not have been traumatising for the individual. That is, something about the individual can influence whether the trauma impacted them via having negative voices, perhaps the consideration of trauma not affecting the person psychologically was discussed.  • Type of trauma for this group potentially being different:   1. Considering that trauma may have been less long lasting types of trauma or perhaps less ongoing at an earlier age. 2. Less negative experiences overall while some traumas experienced may still mean potentially more positive voices.   • Exploration of whether this group has a better wellbeing and experience in life more broadly (“is life kinder to this group?”) meaning could have more positive voice experiences as you have good experiences in your life and the bad experiences are not projected onto voices alone.  • Consideration about the nature of the positive voices heard being related to a higher frequency of voices experienced potentially for this group. That is, if more voices then cannot all be aggressive/bad all the time with a higher rate of frequency, there are spikes where there are more abusive voices but overall more narration and making comments on a day to day thus higher chance of interpreting voices as ok/benevolent?  • Discussions of whether this group may have less improvement in the trial given its mostly positive voices. |

**Supplementary Material G: Full sample information**

The descriptive information in Supplementary Table 5 shows that the full sample of participants (n = 345) had a mean age of 39.6 years old, the majority are of ‘White’ ethnicity, male gender, and have a Schizophrenia and Schizoaffective diagnosis. Supplementary Table 6 also describes in further detail the breakdown of the diagnosis of participants included in the trial and this study.

The indicator variables distribution shown in Table 3 suggests that individuals reported on average moderately high fearful attachment style (M(SD)=5.1(1.9)), with 94.2% reporting experiences of one or more traumatic life events (M(SD)=2.7(1.2)), which were believed to be highly related to voices heard (M(SD)=1.9(1.1)). On average voices were appraised as highly malevolent (M(SD)=11.5(4.3)), omnipotent (M(SD)=11.1(3.6)) and resisted against (M(SD)=19.8(4.7)), while occurrence of benevolent voices and voice engagement was lower (M(SD)=3.3(3.9), (M(SD)=4.3(4.4), respectively). Correlation of indicator variables were found to be weak to moderate (|*r*| range = 0.02 to 0.35) except for a higher association between voice malevolence-omnipotence and benevolence-engagement (|*r*| = 0.61 and 0.70, respectively; see Supplementary Table 7 for further details).

The distal variables shown in Supplementary Table 8 indicate that the full sample shows an expected high severity of voices (M=30.3(4.5)), emotional distress was in the severe range for depression, extremely severe range for anxiety and moderate range for stress, with some impaired in their motivation and pleasure (M=15.8(7.5)) and despite just 5.8% of the sample reporting no traumas only some reported having PTSD symptoms (M=10.6(7.4)).

For an understanding of the characteristics of each latent profile these are described in reference to how their indicator variables distribution is higher/lower relative to the full sample – within the positive SD range: very high: z-score > 1.5 SD from the population mean, high: 1.5 SD > z-score > 1 SD, moderately high: 1 SD > z-score > 0.5 SD, comparable [to the full sample] in a higher range: 0.5 SD > z-score > 0 SD. Within the negative SD range: comparable in a lower range: 0 SD > z-score > -0.5 SD, moderately low: -0.5 SD > z-score > -1 SD, low: -1 SD > z-score > -1.5 SD, very low: z-score < -1.5 SD.

**Supplementary table 5**. *Full sample descriptive characteristics.*

| Characteristics | Mean (SD) |
| --- | --- |
| Age | 39.6 (13.4) |
| Diagnosis* | n (%) |
| F20-F29 – Schizophrenia and Schizoaffective disorders | 301 (87.2%) |
| F31 - Bipolar Affective Disorder | 8 (2.3%) |
| F32.3 - Severe depressive episode with psychotic symptoms | 33 (9.6%) |
| Not available or not applicable | 3 (0.9%) |
| Gender |  |
| Male | 212 (61.4%) |
| Female | 129 (37.4%) |
| Other | 4 (1.2%) |
| Ethnicity background* |  |
| White  Includes White (203), White British Italian (1), White Scottish (2) | 206 (59.7%) |
| Black or mixed Black  Includes Black African (29), Black Caribbean (22), Black Other (10), Black Mixed Ethnicity (11) | 72 (20.9%) |
| South Asian or mixed South Asian  Includes Pakistani (16), Indian (11), Bangladesh (3), Bengali (2), South Asian Mixed Ethnicity (4) | 36 (10.4%) |
| Any Other  Includes East Asian (6), Other (12), Unclear from description (e.g., British) (3), Mixed ethnicity, excluding people included Black or South Asian (9) | 31 (9%) |
| *Note.* * Please see Supplementary Table 6 for full details of diagnosis and Supplementary Table 1 further details of ethnicity for each of the categories in line with the larger AVATAR2 RCT. | |

**Supplementary Table 6.** Table with details of the diagnoses of the full sample (according to ICD-10 codes) from the AVATAR2 DMC report, 2023.

| Diagnosis | Overall (n=345) |
| --- | --- |
| F20 - Schizophrenia | 151 (43.8%) |
| F21 - Schizotypal disorder | 0 (0%) |
| F22 - Persistent delusional disorders | 3 (0.9%) |
| F23 - Acute and transient psychotic disorders | 4 (1.2%) |
| F24 - Induced delusional disorder | 1 (0.3%) |
| F25 - Schizoaffective disorders | 27 (7.8%) |
| F28 - Other nonorganic psychotic disorders | 8 (2.3%) |
| F29 - Unspecified nonorganic psychosis | 107 (31.0%) |
| F31 - Bipolar Affective Disorder | 8 (2.3%) |
| F32.3 - Severe depressive episode with psychotic symptoms | 33 (9.6%) |
| Not available or not applicable | 3 (0.9%) |
|  |  |

**Supplementary table 7.** Correlational matrix with covariance of the indicator variables within the LPA

| Indicator variables | Fearful Attachment - RSQ | Trauma- Mini-TALE | Beliefs about trauma-voice content relatedness - TVAQ | Voice appraisal subscales - BAVQ-R: Malevolence | Voice appraisal subscales - BAVQ-R: Omnipotence | Voice appraisal subscales - BAVQ-R: Resistance | Voice appraisal subscales - BAVQ-R: Benevolence | Voice appraisal subscales - BAVQ-R: Engagement |
| --- | --- | --- | --- | --- | --- | --- | --- | --- |
| Fearful Attachment - RSQ | 1.0 |  |  |  |  |  |  |  |
| Trauma - Mini-TALE | 0.29*** | 1.0 |  |  |  |  |  |  |
| Beliefs about trauma-voice content relatedness - TVAQ | 0.30*** | 0.21** | 1.0 |  |  |  |  |  |
| Voice appraisal subscales - BAVQ-R: Malevolence | 0.17** | 0.12* | 0.19** | 1.0 |  |  |  |  |
| Voice appraisal subscales - BAVQ-R: Omnipotence | 0.14* | 0.06 | 0.20** | 0.61*** | 1.0 |  |  |  |
| Voice appraisal subscales - BAVQ-R: Resistance | 0.21*** | 0.17** | 0.23*** | 0.43*** | 0.35*** | 1.0 |  |  |
| Voice appraisal subscales - BAVQ-R: Benevolence | 0.04 | 0.04 | 0.08 | -0.21*** | 0.04 | -0.18** | 1.0 |  |
| Voice appraisal subscales - BAVQ-R: Engagement | 0.07 | -0.02 | 0.09 | -0.12* | 0.08 | -0.16** | 0.70*** | 1.0 |
| *Note.* *p<0.05, **p<0.01, ***p<0.001. | | | | | | | | |

**Supplementary Table 8.** Distribution of indicator and distal variables in the full sample.

| Indicator variables | Measure^1^ (range) | Mean (SD) | Distal variables | Measure^1^ (range) | Mean (SD) |
| --- | --- | --- | --- | --- | --- |
| Fearful Attachment | RSQ  (1-7; scores of 7 indicate high fearful attachment) | 5.1 (1.9)  [n=341] | Voice hearing severity | PSYRATS-H total subscale  (0-44) | 30.3 (4.5)  [n=345] |
| Trauma | Mini-TALE  (0-4; scores of 4 indicate responding yes for experiencing all trauma types: physical, emotional, sexual abuse and neglect) | 2.7 (1.2)  [n=345]  [5.8% no trauma reported] | PTSD symptoms | ITQ dimensional score  (0-24) | 10.6 (7.4)  [n=345] |
| Beliefs about trauma-voice content relatedness | TVAQ  (0-3; scores of 3 indicate higher voice content-trauma links present) | 1.9 (1.1)  [n=322] | Global Distress | DASS-21: Depression subscale  (normal: 0-9, mild: 10-13, moderate: 14-20, severe: 21-27, extremely severe: 28-42)^2^ | 22.9 (11.3)  Severe  [n=341] |
| Voice appraisal subscale | BAVQ-R: Malevolence  (0-18; higher scores indicate higher appraisal) | 11.5 (4.3)  [n=343] | Global Distress | DASS-21: Anxiety subscale  (normal: 0-7, mild: 8-9, moderate: 10-14, severe: 15-19, extremely severe: 20-42)^2^ | 19.5 (10.3)  Extremely severe  [n=340] |
| Voice appraisal subscales | BAVQ-R: Omnipotence  (0-18; higher scores indicate higher appraisal) | 11.1 (3.6)  [n=341] | Global Distress | DASS-21: Stress subscale  (normal: 0-14, mild: 15-18, moderate: 19-25, severe: 26-33, extremely severe: 34-42)^2^ | 23.0 (10.2)  Moderate  [n=340] |
| Voice appraisal subscales | BAVQ-R: Resistance  (0-27; higher scores indicate higher resistance) | 19.8 (4.7)  [n=342] | Level of motivation and pleasure difficulties | CAINS: motivation-pleasure (MAP) subscale  (0-36; cut-off score considered was 17 for the MAP (Li et al., 2018). | 15.8 (7.5)  [n=345] |
| Voice appraisal subscales | BAVQ-R: Benevolence  (0-18; higher scores indicate higher appraisal) | 3.3 (3.9)  [n=343] |  |  |  |
| Voice appraisal subscales | BAVQ-R: Engagement  (0-24; higher scores indicate higher engagement) | 4.3 (4.4)  [n=342] |  |  |  |
| *Note.* ^1^see Supplementary Material B for full measures information, including total scores and cut off points.  ^2^DASS-21 is scored via doubling summed scores for each subscale given it is the short form for the scale. | | | | | |

**Supplementary Material H: Graphical representation of latent profiles and their indicator variables distribution**

For each LP, the distribution of indicator variables of attachment, trauma presence and its association with voice content and the different voice appraisals subscales are presented graphically in Supplementary Figure 7. Since indicator variables have different continuous scales which cannot be directly compared, standardised mean z-scores are used to compare the distribution of variables across LPs relative to the full sample. A brief description of each profile’s indicator variable distribution can be seen below the figure, where within the positive SD range from the population mean: Very High: z-score > 1.5 SD from the population mean, High: = 1.5 SD > z-score > 1 SD, Moderately high: 1 SD > z-score > 0.5 SD, Comparable [to the full sample] in a higher range: 0.5 SD > z-score > 0 SD; within the negative SD range from the population mean: Comparable [to the full sample] in a lower range: 0 SD > z-score > -0.5 SD, Moderately low: -0.5 SD > z-score > -1 SD; Low: -1 SD > z-score > -1.5 SD, Very Low: z-score < -1.5 SD.

**Supplementary Figure *4.*** *A graphical representation of the Latent Profiles (LP) and their indicator variables distributions.*


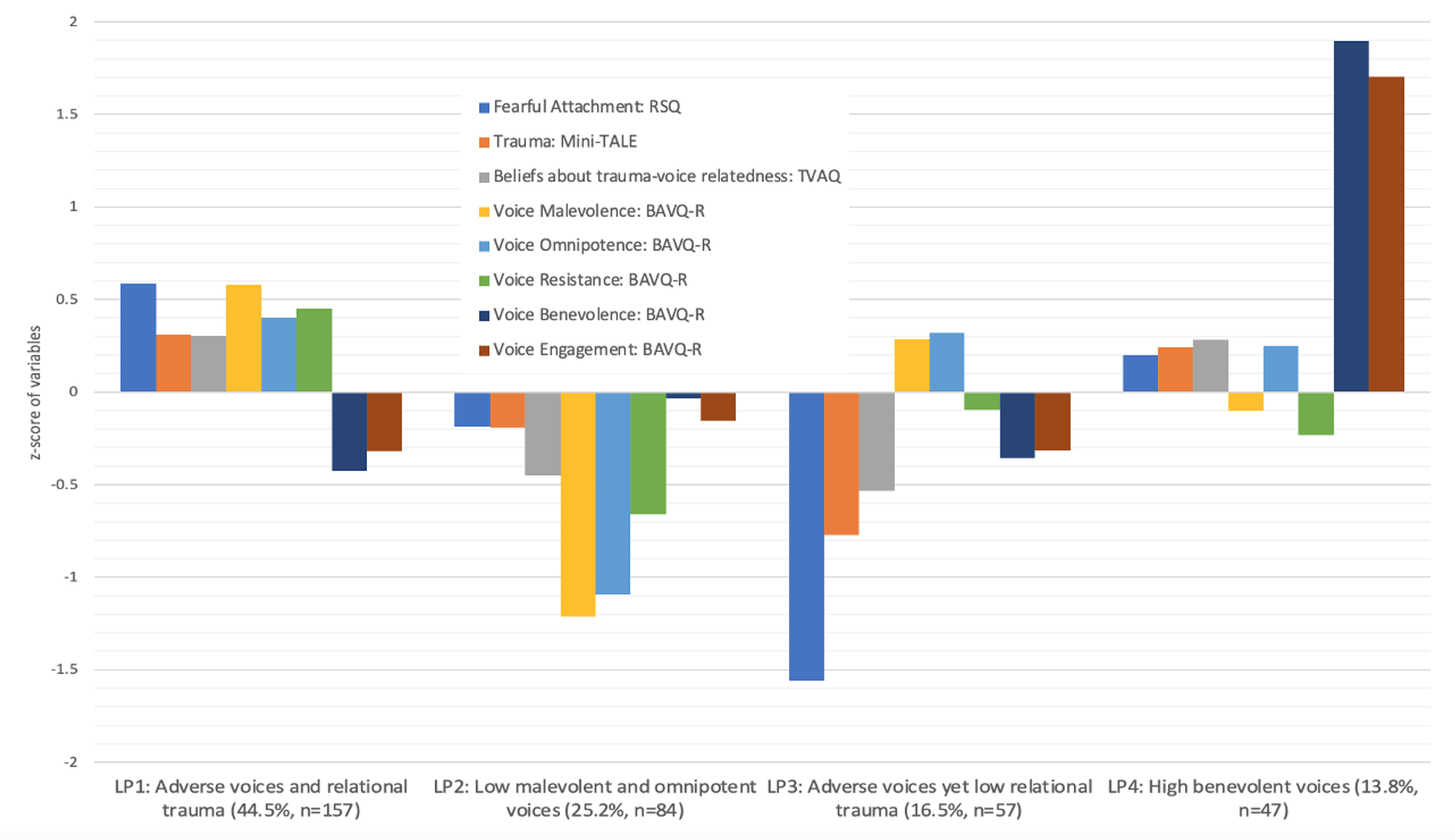

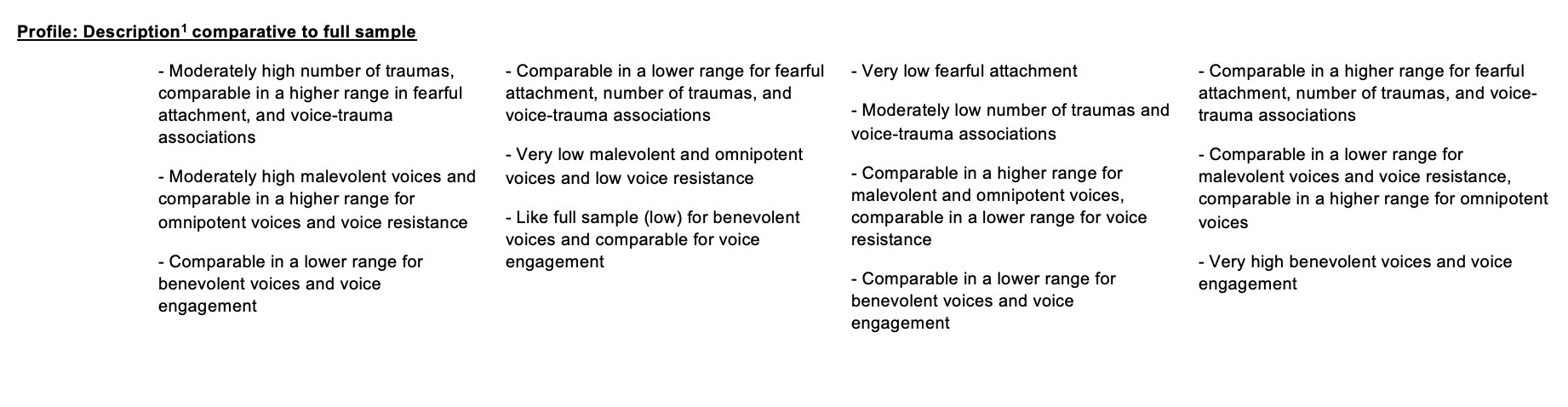


#### Supplementary Material I: Further details of post-LPA analyses results for demographic covariates and distal clinical outcomes and their association with profiles

All descriptive statistics are taken using the exact probability of belonging to each LP meaning there will be some variability in reported means as compared to those in the statistical tests since these account for error in profile classification using the bias-adjusted R3STEP and BCH procedures. Missing data was handled by multiple imputation for the statistical analyses.

**Demographic covariates’ association with profiles**

The descriptive statistics of age, gender, and ethnicity on average show age seems to be within the late 30s to early 40s years old range with a higher percentage of males across profiles. The descriptive distribution of ethnicity is similar across profiles with a majority of ‘White’ ethnicity followed by ‘Black or mixed Black’ ethnicity. ‘South Asian or mixed South Asian’ ethnicity is more highly represented in LP1 ‘Adverse voice and relational trauma’ and LP3 ‘Adverse voices yet low relational trauma’ and is lowest in LP2 ‘Low malevolent and omnipotent voices’ and LP4 ‘High benevolent voices’. ‘Any other’ ethnicity is more highly represented in LP4 ‘High benevolent voices’ as compared to a much lower percentage across other profiles.

The multinomial logistic regressions considering the associations of gender, age and ethnicity with each profile used LP1 ‘Adverse voices and relational trauma’ as the reference profile (as the largest sample size) compared against can be seen in Supplementary Table 9. The gender demographic was treated as binary (i.e., ‘male’ and ‘female and other genders’) due to the extremely small numbers within ‘Other’ genders resulting in underpowered comparisons. Only two statistically significant associations between demographics and profile membership were observed as seen in Supplementary Table 9, indicating differences in gender and ethnicity.

**Supplementary Table 9.** Multinomial logistic regression analyses comparing demographic covariates associated with profile membership.

| Profile compared to Reference: LP1 ‘Adverse voices and relational trauma’ | Demographic covariates | n | Odds ratio (95% CI) | p-value |
| --- | --- | --- | --- | --- |
| LP2 ‘Low malevolent and omnipotent voices’ | Age | | 0.982 (0.959; 1.006) | 0.136 |
| LP3 ‘Adverse voices yet low relational trauma’ |  |  | 0.973 (0.945; 1.002) | 0.065 |
| LP4 ‘High benevolent voices’ |  |  | 0.974 (0.946; 1.003) | 0.073 |
|  | Gender |  |  |  |
| LP2 ‘Low malevolent and omnipotent voices’ | Male | 58 | Ref | Ref |
|  | Female and other genders | 25 | 0.472 (0.247; 0.901) | 0.023* |
| LP3 ‘Adverse voices yet low relational trauma’ | Male | 40 | Ref | Ref |
|  | Female and other genders | 17 | 0.45 (0.198; 1.022) | 0.056 |
| LP4 ‘High benevolent voices’ | Male | 29 | Ref | Ref |
|  | Female and other genders | 17 | 0.676 (0.321; 1.425) | 0.304 |
|  | Ethnicity |  |  |  |
| LP2 ‘Low malevolent and omnipotent voices’ | White | 51 | Ref | Ref |
|  | Black & mixed Black | 19 | 1.385 (0.637; 3.011) | 0.411 |
|  | South Asian & mixed South Asian | 6 | 0.499 (0.157; 1.585) | 0.238 |
|  | Any Other | 8 | 2.15 (0.613; 7.533) | 0.232 |
| LP3 ‘Adverse voices yet low relational trauma’ | White | 34 | Ref | Ref |
|  | Black & mixed Black | 12 | 1.333 (0.524; 3.396) | 0.546 |
|  | South Asian & mixed South Asian | 8 | 1.073 (0.357; 3.224) | 0.901 |
|  | Any Other | 3 | 1.137 (0.186; 6.948) | 0.89 |
| LP4 ‘High benevolent voices’ | White | 20 | Ref | Ref |
|  | Black & mixed Black | 12 | 2.251 (0.92; 5.508) | 0.076 |
|  | South Asian & mixed South Asian | 3 | 0.647 (0.139; 3.008) | 0.578 |
|  | Any Other | 12 | 8.78 (2.75; 28.032) | <0.001*** |
| *Note.* *p<0.05 **p<0.01 ***p<0.001 | | | | |

#### Clinical outcomes’ association with profiles

Supplementary Figure 8 graphically represents and summarises the standardised mean z-scores relative to the full sample for all clinical outcome variables, given such variables have different continuous scales which cannot be directly compared. Overall, LP1 ‘Adverse voices and relational trauma’ has the highest scores across all clinical outcomes across profiles, which is higher than the full sample, while LP2 ‘Low malevolent and omnipotent voices’ has the lowest scores across outcomes, lower than the full sample scores, albeit for PTSD where LP3 ‘Adverse voices yet low relational trauma’ has the lowest score. LP3 is second highest on voices severity followed by LP4 ‘High benevolent voices’. LP4 ‘High benevolent voices’ has the second highest scores in PTSD, global distress, and close second highest score with LP3 ‘Adverse voices yet low relational trauma’ on difficulties with motivation and pleasure.

In these analyses, gender and ethnicity variables were binary dummy variables (i.e., male/female and other genders, and white/not-white ethnicities, including ‘Black & mixed Black’, ‘South Asian & mixed South Asian’ and ‘Any Other’) due to low-powered distribution of separate categories. Analyses for considering association of latent profiles with clinical outcomes while accounting for demographic covariates focused on the profile-specific intercept regressions, seen in Supplementary Table 10. All profiles are found to have a unique influence on outcomes beyond the demographic covariates effect. Then omnibus chi-square tests compare these profile-specific intercepts to see if LPs have significantly different influences, seen in Supplementary Table 10 and 11. These indicated that latent profiles were not significantly different in their associations with outcomes of PTSD symptoms and difficulties with motivation and pleasure when controlling for covariates. Alternatively, belonging to different profiles had a significant profile-specific association for outcomes of voice severity and global distress (depression, anxiety, and stress) after controlling for the covariates’ influences. For the chi-square tests showing significance, then the pairwise z-tests, in Supplementary Table 11, can be further considered, as is the case for clinical outcomes of voice severity and global distress below. While for PTSD and motivation and pleasure difficulties outcomes descriptive statistics are considered only below.

*Voice severity:* LP1 ‘Adverse voices and relational trauma’ and LP3 ‘Adverse voices yet low relational trauma’ are not significantly different, with the highest average scores in this outcome, yet they are both significantly different from the other profiles (LP2 ‘Low malevolent and omnipotent voices’ and LP4 ‘High benevolent voices’) which were not significantly different between themselves in their association with the lower voice severity scores in such profiles.

*Emotional distress:* LP1 ‘Adverse voices and relational trauma’ with the highest average score of severe depression, is significantly different from LP2 ‘Low malevolent and omnipotent voices’ with the lowest average score of moderate depression, and LP4 ‘High benevolent voices’, with the second highest average score of extremely severe depression. Also, LP1 ‘Adverse voices and relational trauma’ with the highest average score of extremely severe anxiety, is significantly different from LP2 ‘Low malevolent and omnipotent voices’ with the lowest average score of moderate anxiety. Finally, LP1 ‘Adverse voices and relational trauma’ with the highest average score of severe stress, is significantly different from LP2 ‘Low malevolent and omnipotent voices’ with the lowest average score of mild stress.

*PTSD symptoms:* LP1 ‘Adverse voices and relational trauma’ and LP4 ‘High benevolent voices’ show similar high scores and are both relatively higher than the full sample. The other LPs are substantially lower than the full sample where LP3 ‘Adverse voices yet low relational trauma’ has the lowest PTSD score.

*Motivation and pleasure difficulties:* LP1 Adverse voices and relational trauma’, followed by LP3 ‘Adverse voices yet low relational trauma’ and LP4 ‘High benevolent voices’ had similar relatively higher than the full sample average score, while LP2 ‘Low malevolent and omnipotent voices’ showed the lowest score, below the full sample average in difficulties with motivation and pleasure.


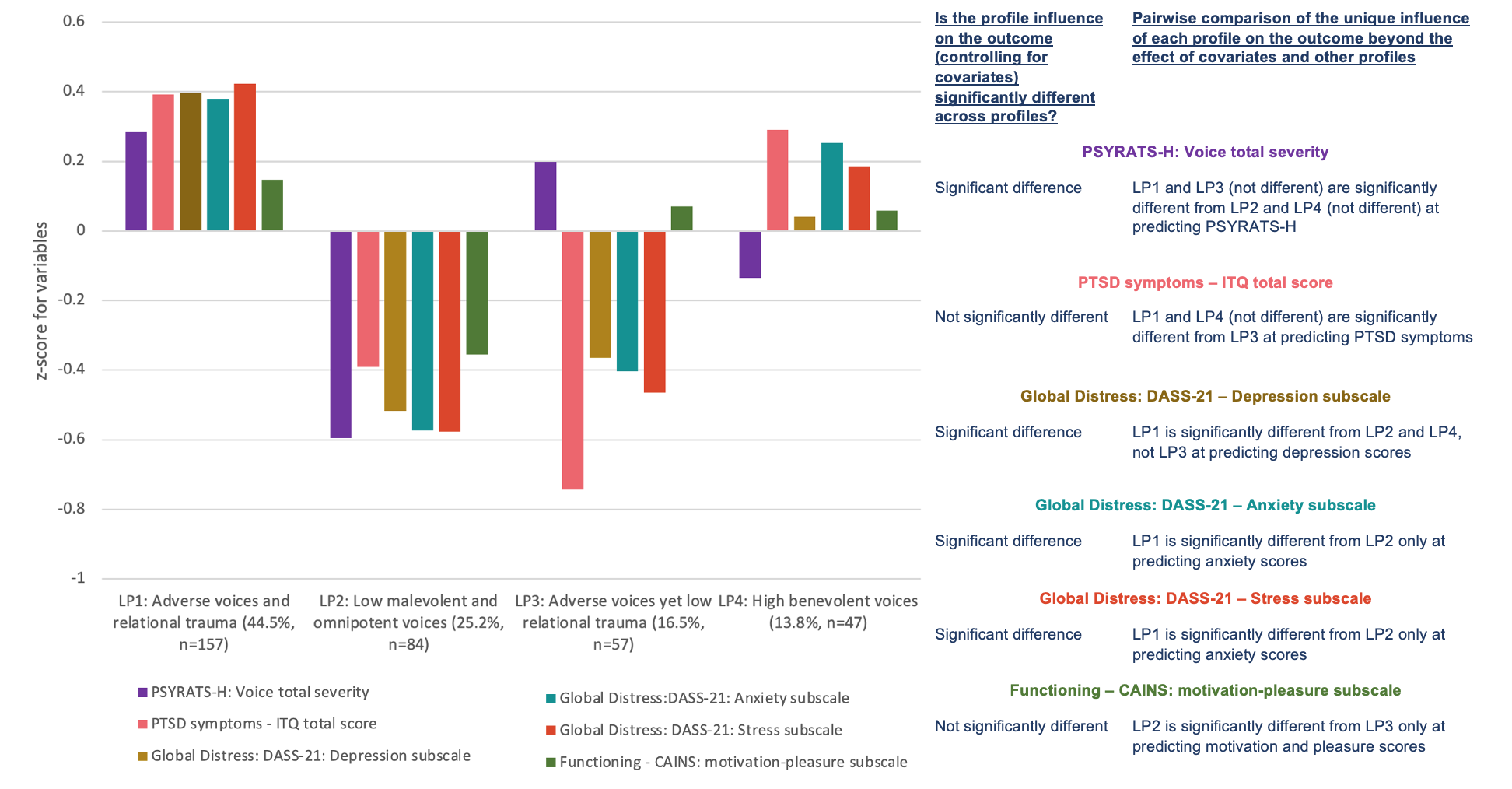
**Supplementary Figure 5.** A graphical representation of the Latent Profiles (LP) and their distal outcome variables distributions.

**Supplementary Table *10***. *Profile-specific intercept regression statistics and omnibus chi-square comparisons depicting the influence of the latent profiles on each distal outcome beyond the influence of the demographic covariates.*

| Distal outcome variable with the influence of demographic covariates binary gender (male/female and other genders), ethnicity (white/not-white ethnicities), age included | Profile | Comparative statistic test F, Confidence interval (95%CI), p-value^1^ | Comparative statistic test of omnibus chi-square, X^2^ for intercepts and p-value^2^ |
| --- | --- | --- | --- |
| Total severity of voice: PSYRATS-H total subscale | LP1 ‘Adverse voices and relational trauma’ | F=30.6 (95%CI: 28.3, 32.7), p<0.001 | X^2^(3)=10.4, p=0.015* |
|  | LP2 ‘Low malevolent and omnipotent voices’ | F=26.6 (95%CI: 23.9, 29.1), p<0.001 |  |
|  | LP3‘Adverse voices yet low relational trauma’ | F=31.5 (95%CI: 28.1, 35.6), p<0.001 |  |
|  | LP4‘High benevolent voices’ | F=27.1 (95%CI: 24.6, 29.5), p<0.001 |  |
| PTSD symptoms: ITQ dimensional score | LP1‘Adverse voices and relational trauma’ | F=12.5 (95%CI: 8.8, 16.2), p<0.001 | X^2^(3)=7.2, p=0.066 |
|  | LP2 ‘Low malevolent and omnipotent voices’ | F=9.8 (95%CI: 6.4, 13.1), p<0.001 |  |
|  | LP3‘Adverse voices yet low relational trauma’ | F=5.8 (95%CI: 0.3, 13.5), p<0.001 |  |
|  | LP4 High benevolent voices’ | F=13.9 (95%CI: 9.7, 18.0), p<0.001 |  |
| Level of global distress:  DASS-21 depression subscale | LP1‘Adverse voices and relational trauma’ | F=27.2 (95%CI: 21.7, 32.8), p<0.001 | X^2^(3)=8.4, p=0.038* |
|  | LP2 ‘Low malevolent and omnipotent voices’ | F=16.3 (95%CI: 10.8, 21.8), p<0.001 |  |
|  | LP3‘Adverse voices yet low relational trauma’ | F=19.0 (95%CI: 10.7, 27.3), p<0.001 |  |
|  | LP4‘High benevolent voices’ | F=17.7 (95%CI: 11.9, 23.5), p<0.001 |  |
| Level of global distress:  DASS-21 anxiety subscale | LP1‘Adverse voices and relational trauma’ | F=25.8 (95%CI: 20.5, 31.0), p<0.001 | X^2^(3)=10.2, p=0.017* |
|  | LP2‘Low malevolent and omnipotent voices’ | F=13.8 (95%CI: 9.3, 18.6), p<0.001 |  |
|  | LP3‘Adverse voices yet low relational trauma’ | F=18.9 (95%CI: 12.8, 24.9), p<0.001 |  |
|  | LP4‘High benevolent voices’ | F=17.8 (95%CI: 11.5, 24.1), p<0.001 |  |
| Level of global distress:  DASS-21 stress subscale | LP1‘Adverse voices and relational trauma’ | F=27.3 (95%CI: 22.5, 32.1), p<0.001 | X^2^(3)=10.3, p=0.016* |
|  | LP2‘Low malevolent and omnipotent voices’ | F=17.1 (95%CI: 13.0, 21.2), p<0.001 |  |
|  | LP3‘Adverse voices yet low relational trauma’ | F=17.6 (95%CI: 7.9, 27.4), p<0.001 |  |
|  | LP4‘High benevolent voices’ | F=23.1 (95%CI: 17.6, 28.5), p<0.001 |  |
| Level of motivation and pleasure difficulties: CAINS motivation-pleasure subscale | LP1‘Adverse voices and relational trauma’ | F=15.4 (95%CI: 11.5, 19.2), p<0.001 | X^2^(3)=6.7, p=0.083 |
|  | LP2‘Low malevolent and omnipotent voices’ | F=10.9 (95%CI: 6.9, 15.0), p<0.001 |  |
|  | LP3‘Adverse voices yet low relational trauma’ | F=19.0 (95%CI: 12.5, 27.6), p<0.001 |  |
|  | LP4‘High benevolent voices’ | F=11.2 (95%CI: 6.3, 16.5), p<0.001 |  |
| *Note:*^1^ The F statistic represents the profile-specific intercept multinomial regressions representing the influence of the latent profile on the outcome mean beyond the influence of covariates controlled for. Confidence intervals were acquired through bias corrected bootstrapping for severity of voices, PTSD symptoms, and motivation and pleasure scales, while it was acquired normally for level of global distress given multiple imputation does not allow for bootstrapping procedure in Mplus.  *^2^* The omnibus Wald chi-square tested for the equivalence of each distal outcome mean intercept difference compared across profiles while controlling for covariate influence.  *p<0.05 **p<0.01 ***p<0.001 | | | |

**Supplementary Table 11 description for ease of reading:** Each level displays the comparison across the vertical LP with the horizontal LP for each distal outcome, where what is reported is: the z-test on the pairwise difference between the LPs distal outcome intercepts (i.e., from the regression intercepts) to see if each profile influences the outcome controlling for the covariates, the bias-corrected bootstrapping developed confidence intervals (95% CI), and the p value significance.

Intercept estimate represents the mean of a distal outcome in each profile accounting for covariates and other profiles. Thus, showing the influence of the LP on the outcome beyond the influence of covariates.

**Supplementary Table 11.** Table with equivalence omnibus chi-square and pairwise z-tests comparing profile-specific intercepts indicating association of profile membership differentially to each clinical presentation outcome while controlling for demographic covariates.

| Distal outcome variable | Profile | Outcome mean estimate | Profile regression intercept estimate | LP1 ‘Adverse voices and relational trauma’ | LP2 ‘Low malevolent and omnipotent voices’ | LP3 ‘Adverse voices yet low relational trauma’ | Overall X^2^ for outcome intercept |
| --- | --- | --- | --- | --- | --- | --- | --- |
| Total severity of voice: PSYRATS-H total subscale | LP1 ‘Adverse voices and relational trauma’ | 31.76 | 30.59 | x |  |  | X^2^(3)=10.44, p=0.015* |
|  | LP2 ‘Low malevolent and omnipotent voices’ | 27.27 | 26.58 | -4.01 (95% CI: -7.55, -0.34)  p=0.021* | x |  |  |
|  | LP3 ‘Adverse voices yet low relational trauma’ | 31.36 | 31.51 | 0.92 (95% CI: -3.51, 5.57)  p=0.639 | -4.93 (95% CI:-10.08, -0.39)  p=0.018* | x |  |
|  | LP4 ‘High benevolent voices’ | 29.60 | 27.07 | -3.52 (95% CI: -6.81, -0.24)  p=0.03* | -0.49 (95% CI: -4.26, 3.17)  p=0.78 | -4.44 (95% CI: -9.1, -0.04)  p=0.024* |  |
| PTSD symptoms: ITQ dimensional score | LP1 ‘Adverse voices and relational trauma’ | 14.02 | 12.45 | x |  |  | X^2^(3)=7.19, p=0.066 |
|  | LP2 ‘Low malevolent and omnipotent voices’ | 7.50 | 9.77 | -2.68 (95% CI: -7.97, 2.33)  p=0.274 | x |  |  |
|  | LP3 ‘Adverse voices yet low relational trauma’ | 4.11 | 5.76 | -6.69 (95% CI: -13.72, 1.52)  p=0.042* | 4.01 (95% CI: -4.43, 10.84)  p=0.208 | x |  |
|  | LP4 ‘High benevolent voices’ | 12.82 | 13.92 | 1.47 (95% CI: -4.30, 7.02)  p=0.584 | -4.14 (95% CI: -9.61, 1.19)  p=0.104 | 8.16 (95% CI: -0.56, 15.08)  p=0.015* |  |
| Level of global distress:  DASS-21 depression subscale | LP1 ‘Adverse voices and relational trauma’ | 28.24 | 27.24 | x |  |  | X^2^(3)=8.42, p=0.038* |
|  | LP2 ‘Low malevolent and omnipotent voices’ | 16.49 | 16.30 | -10.94 (95% CI: -19.0, -2.88)  p=0.008** | x |  |  |
|  | LP3 ‘Adverse voices yet low relational trauma’ | 17.93 | 19.0 | -8.25 (95% CI: -18.96, 2.13)  p=0.119 | -2.70 (95% CI: -13.03, 7.64)  p=0.609 | x |  |
|  | LP4 ‘High benevolent voices’ | 23.33 | 17.70 | -9.54 (95% CI: -17.68, -1.41)  p=0.022* | -1.40 (95% CI: -9.54, 6.74)  p=0.736 | -1.30 (95% CI: -11.52, 8.93)  p=0.804 |  |
| Level of global distress:  DASS-21 anxiety subscale | LP1 ‘Adverse voices and relational trauma’ | 24.12 | 25.75 | x |  |  | X^2^(3)=10.24, p=0.017* |
|  | LP2 ‘Low malevolent and omnipotent voices’ | 12.96 | 13.81 | -11.94 (95% CI: -19.30, -4.57)  p=0.001** | x |  |  |
|  | LP3 ‘Adverse voices yet low relational trauma’ | 14.62 | 18.85 | -6.90 (95% CI: -15.25, 1.44)  p=0.105 | -5.03 (95% CI: -13.07, 3.0)  p=0.219 | x |  |
|  | LP4 ‘High benevolent voices’ | 22.2 | 17.81 | -7.94 (95% CI: -16.27, 0.39)  p=0.062 | -3.99 (95% CI: -12.05, 4.06)  p=0.331 | -1.04 (95% CI: -9.84, 7.77)  p=0.817 |  |
| Level of global distress:  DASS-21 stress subscale | LP1 ‘Adverse voices and relational trauma’ | 28.19 | 27.06 | x |  |  | X^2^(3)=10.30, p=0.016* |
|  | LP2 ‘Low malevolent and omnipotent voices’ | 16.53 | 17.07 | -10.22 (95% CI: -16.74, -3.70)  p=0.002** | x |  |  |
|  | LP3 ‘Adverse voices yet low relational trauma’ | 17.24 | 17.61 | -9.68 (95% CI: -20.92, 1.56)  p=0.091 | -0.54 (95% CI: -11.43, 10.35) p=0.923 | x |  |
|  | LP4 ‘High benevolent voices’ | 24.96 | 23.06 | -4.23 (95% CI: -11.64, 3.18)  p=0.264 | -5.99 (95% CI: -12.97, 0.99)  p=0.092 | 5.45 (95% CI: -5.80, 16.71)  p=0.342 |  |
| Level of motivation and pleasure difficulties: CAINS motivation-pleasure subscale | LP1 ‘Adverse voices and relational trauma’ | 17.12 | 15.36 | x |  |  | X^2^(3)=6.67, p=0.083 |
|  | LP2 ‘Low malevolent and omnipotent voices’ | 12.83 | 10.91 | -4.45 (95% CI: -10.20, 1.20)  p=0.109 | x |  |  |
|  | LP3 ‘Adverse voices yet low relational trauma’ | 16.50 | 18.98 | 3.62 (95% CI: -4.28,12.93)  p=0.318 | -8.07 (95% CI:-17.81, -0.03)  p=0.028* | x |  |
|  | LP4 ‘High benevolent voices’ | 16.27 | 11.24 | -4.12 (95% CI: -10.60, 2.58)  p=0.186 | -0.33 (95% CI: -6.84, 6.20)  p=0.917 | -7.74 (95% CI:-17.39, 0.69)  p=0.46 |  |
| *Note. For all level of global distress subscales that required a multiple imputation procedure to handle missing outcomes the distal mean is averaged over 50 data sets only.*  *p<0.05 **p<0.01 ***p<0.001 | | | | | | | |

**References**

Airey, N. D., Taylor, C. D., Vikram, A., & Berry, K. (2023). Trauma measures for use with psychosis populations: A systematic review of psychometric properties using COSMIN. *Psychiatry Research*, *323*(2023), 1–13. doi:10.1016/j.psychres.2023.115163

Andrew, E. M., Gray, N. S., & Snowden, R. J. (2008). The relationship between trauma and beliefs about hearing voices: a study of psychiatric and non-psychiatric voice hearers. *Psychological medicine*, *38*(10), 1409–1417. doi: 10.1017/S003329170700253X

Asparouhov, T., & Muthén, B. (2012). Using Mplus TECH11 and TECH14 to test the number of latent classes. *Mplus web notes*, *14*, 1–19. Available at: <http://www.statmodel.com/examples/webnotes/webnote14.pdf>

Asparouhov, T., & Muthén, B. (2014). Auxiliary variables in mixture modeling: Three-step approaches using Mplus. *Mplus web notes*, *15*, 1-51. Available at: <https://www.statmodel.com/download/webnotes/webnote15.pdf>

Asparouhov, T., & Muthén, B. (2021). Auxiliary variables in mixture modeling: Using the BCH method in Mplus to estimate a distal outcome model and an arbitrary secondary model. *Mplus web notes*, *21*, 1-80. Available at: <https://www.statmodel.com/examples/webnotes/webnote21.pdf>

Bakk, Z., & Kuha, J. (2020). Relating latent class membership to external variables: An overview. *British Journal of Mathematical and Statistical Psychology*, *74*(2), 340–362. doi:10.1111/bmsp.12227

Bakk, Z., & Vermunt, J. K. (2016). Robustness of stepwise latent class modeling with continuous distal outcomes. *Structural equation modeling: a multidisciplinary journal*, *23*(1), 20–31. doi:10.1080/10705511.2014.955104

Bakk, Z., Tekle, F. B., & Vermunt, J. K. (2013). Estimating the association between latent class membership and external variables using bias-adjusted three-step approaches. *Sociological methodology*, *43*(1), 272–311. doi:10.1177/0081175012470644

Bolck, A., Croon, M., & Hagenaars, J. (2004). Estimating latent structure models with categorical variables: One-step versus three-step estimators. *Political analysis*, *12*(1), 3–27. doi:10.1093/pan/mph001

Bray, B. C., Lanza, S. T., & Tan, X. (2015). Eliminating bias in classify-analyze approaches for latent class analysis. *Structural equation modeling: a multidisciplinary journal*, *22*(1), 1–11. doi:10.1080/10705511.2014.935265.

Chadwick, P., Lees, S., & Birchwood, M. A. X. (2000). The revised beliefs about voices questionnaire (BAVQ–R). *The British Journal of Psychiatry*, *177*(3), 229–232. doi:10.1192/bjp.177.3.229

Clark, S. L., & Muthén, B. (2009). Relating latent class analysis results to variables not included in the analysis. Available at: https://www.statmodel.com/download/relatinglca.pdf

Cloitre, M., Hyland, P., Prins, A., & Shevlin, M. (2021). The international trauma questionnaire (ITQ) measures reliable and clinically significant treatment-related change in PTSD and complex PTSD. *European Journal of Psychotraumatology*, *12*(1), 1–12. doi:10.1080/20008198.2021.1930961

Cloitre, M., Shevlin, M., Brewin, C. R., Bisson, J. I., Roberts, N. P., Maercker, A., ... & Hyland, P. (2018). The International Trauma Questionnaire: Development of a self‐report measure of ICD‐11 PTSD and complex PTSD. *Acta Psychiatrica Scandinavica*, *138*(6), 536–546. doi:10.1111/acps.12956

Dalmaijer, E. S., Nord, C. L., & Astle, D. E. (2022). Statistical power for cluster analysis. *BMC bioinformatics*, *23*(1), 1–28. doi:10.1186/s12859-022-04675-1

Degnan, A., Berry, K., Crossley, N., & Edge, D. (2023). Social network characteristics of Black African and Caribbean people with psychosis in the UK. *Journal of psychiatric research*, 161, 62–70. doi:10.1016/j.jpsychires.2023.03.006

Devonport, T. J., Ward, G., Morrissey, H., Burt, C., Harris, J., Burt, S., ... & Nicholls, W. (2022). A Systematic Review of Inequalities in the Mental Health Experiences of Black African, Black Caribbean and Black-mixed UK Populations: Implications for Action. *Journal of Racial and Ethnic Health Disparities*, 1–13. doi:10.1007/s40615-022-01352-0

Drake, R., Haddock, G., Tarrier, N., Bentall, R., & Lewis, S. (2007). The Psychotic Symptom Rating Scales (PSYRATS): their usefulness and properties in first episode psychosis. *Schizophrenia research*, *89*(1-3), 119–122. doi:10.1016/j.schres.2006.04.024

Edge, D., Degnan, A., Cotterill, S., Berry, K., Drake, R., Baker, J., ... & Abel, K. (2016). Culturally-adapted Family Intervention (CaFI) for African-Caribbeans diagnosed with schizophrenia and their families: a feasibility study protocol of implementation and acceptability. *Pilot and feasibility studies*, *2*(1), 1–14. doi:10.1186/s40814-016-0070-2

Fornells‐Ambrojo, M., Johns, L., Onwumere, J., Garety, P., Milosh, C., Iredale, C., ... & Jolley, S. (2017). Experiences of outcome monitoring in service users with psychosis: findings from an Improving Access to Psychological Therapies for people with Severe Mental Illness (IAPT‐SMI) demonstration site. *British Journal of Clinical Psychology*, *56*(3), 253–272. doi:10.1111/bjc.12136

Garber, A. C. (2021). *BCH Two-step Auxiliary Variable Integration with Mplus Automation*. Available at: <https://psyarxiv.com/wmfcj/download?format=pdf>

Garety, P., Edwards, C. J., Ward, T., Emsley, R., Huckvale, M., McCrone, P., ... & Craig, T. (2021). Optimising AVATAR therapy for people who hear distressing voices: study protocol for the AVATAR2 multi-centre randomised controlled trial. *Trials*, *22*(1), 1–17. doi:10.1186/s13063-021-05301-w

Geiser, C. (2013). *Data Analysis with Mplus*. Guilford, New York.

Griffin, D. W., & Bartholomew, K. (1994). Models of the self and other: Fundamental dimensions underlying measures of adult attachment. *Journal of Personality and Social Psychology*, *67*(3), 430–445. doi:10.1037/0022-3514.67.3.430

Haddock, G., McCarron, J., Tarrier, N., & Faragher, E. B. (1999). Scales to measure dimensions of hallucinations and delusions: the psychotic symptom rating scales (PSYRATS). *Psychological medicine*, *29*(4), 879–889. doi:10.1017/s0033291799008661

Henry, J. D., & Crawford, J. R. (2005). The short‐form version of the Depression Anxiety Stress Scales (DASS‐21): Construct validity and normative data in a large non‐clinical sample. *British journal of clinical psychology*, *44*(2), 227–239. doi:10.1348/014466505X29657

Horan, W. P., Kring, A. M., Gur, R. E., Reise, S. P., & Blanchard, J. J. (2011). Development and psychometric validation of the Clinical Assessment Interview for Negative Symptoms (CAINS). *Schizophrenia research*, *132*(2-3), 140–145.

Hyland, P., Shevlin, M., Brewin, C. R., Cloitre, M., Downes, A. J., Jumbe, S., ... & Roberts, N. P. (2017). Validation of post‐traumatic stress disorder (PTSD) and complex PTSD using the International Trauma Questionnaire. *Acta Psychiatrica Scandinavica*, *136*(3), 313–322. doi:10.1111/acps.12771

Islam, Z., Rabiee, F., & Singh, S. P. (2015). Black and minority ethnic groups’ perception and experience of early intervention in psychosis services in the United Kingdom. *Journal of Cross-Cultural Psychology*, *46*(5), 737–753. doi:10.1177/0022022115575737

Kring, A. M., Gur, R. E., Blanchard, J. J., Horan, W. P., & Reise, S. P. (2013). The clinical assessment interview for negative symptoms (CAINS): final development and validation. *American journal of psychiatry*, *170*(2), 165–172. doi:10.1176/appi.ajp.2012.12010109

Lee, J., Lee, E. H., & Moon, S. H. (2019). Systematic review of the measurement properties of the Depression Anxiety Stress Scales–21 by applying updated COSMIN methodology. *Quality of Life Research*, *28*, 2325–2339. doi:10.1007/s11136-019-02177-x

Lenoir, R., & Wong, K. K. Y. (2023). Impact of the COVID-19 pandemic on young people from black and mixed ethnic groups’ mental health in West London: a qualitative study. *BMJ open*, *13*(5), e071903. doi:10.1136/bmjopen-2023-071903

Li, Y., Li, W. X., Zou, Y. M., Yang, Z. Y., Xie, D. J., Yang, Y., ... & Chan, R. C. (2018). Revisiting the persistent negative symptoms proxy score using the Clinical Assessment Interview for Negative Symptoms. *Schizophrenia research*, *202*, 248–253. doi:10.1016/j.schres.2018.07.005

Lo, Y., Mendell, N. R., & Rubin, D. B. (2001). Testing the number of components in a normal mixture. *Biometrika*, *88*(3), 767–778. doi:10.1093/biomet/88.3.767

Masyn, K. E. (2013). Latent class analysis and finite mixture modeling. In T. D. Little (Ed.), *The oxford handbook of quantitative methods: Statistical Analysis*, (Vol 2, pp. 551–611). Oxford University Press.

McLarnon, M. J., & O’Neill, T. A. (2018). Extensions of auxiliary variable approaches for the investigation of mediation, moderation, and conditional effects in mixture models. *Organizational Research Methods*, *21*(4), 955–982. doi:10.1177/1094428118770731

Morin, A. J., McLarnon, M. J., & Litalien, D. (2020). Mixture modeling for organizational behavior research. In Y. Griep, & S. D. Hanssen (Eds.), *Handbook on the temporal dynamics of organizational behavior* (pp. 351–379). Edward Elgar Publishing.

Ng, F., Trauer, T., Dodd, S., Callaly, T., Campbell, S., & Berk, M. (2007). The validity of the 21-item version of the Depression Anxiety Stress Scales as a routine clinical outcome measure. *Acta neuropsychiatrica*, *19*(5), 304–310. doi: 10.1111/j.1601-5215.2007.00217.x

Nylund-Gibson, K., Grimm, R. P., & Masyn, K. E. (2019). Prediction from latent classes: A demonstration of different approaches to include distal outcomes in mixture models. *Structural equation modeling: A multidisciplinary Journal*, *26*(6), 967–985. doi:10.1080/10705511.2019.1590146

Nylund, K. L., Asparouhov, T., & Muthén, B. O. (2007). Deciding on the number of classes in latent class analysis and growth mixture modeling: A Monte Carlo simulation study. *Structural equation modeling: A multidisciplinary Journal*, *14*(4), 535–569. doi:10.1080/10705510701575396

Office for National Statistics (ONS). (2022). *ONS website, statistical bulletin, Ethnic group, England and Wales: Census 2021*. Available at: <https://www.ons.gov.uk/peoplepopulationandcommunity/culturalidentity/ethnicity/bulletins/ethnicgroupenglandandwales/census2021>).

Ratcliff, K., Farhall, J., & Shawyer, F. (2011). Auditory hallucinations: a review of assessment tools. *Clinical Psychology & Psychotherapy*, *18*(6), 524–534. doi:10.1002/cpp.729

Raykov, T., & Marcoulides, G. A. (2004). Using the delta method for approximate interval estimation of parameter functions in SEM. *Structural Equation Modeling*, *11*(4), 621–637. doi:10.1207/s15328007sem1104_7

Rost, J. (2003). Latent class analysis. In R. Fernandez-Ballesteros (Ed*.), Encyclopedia of psychological assessment* (Vol. 1, pp. 539–543). Sage.

Schmitt, D. P., Alcalay, L., Allensworth, M., Allik, J., Ault, L., Austers, I., ... & ZupanÈiÈ, A. (2004). Patterns and universals of adult romantic attachment across 62 cultural regions: Are models of self and of other pancultural constructs?. *Journal of Cross-Cultural Psychology*, *35*(4), 367–402. doi:10.1177/0022022104266105

Shawyer, F., Mackinnon, A., Farhall, J., Mullen, P., Sims, E., Blaney, S., ... & Copolov, D. (2003). Risk factors for compliance with harmful command hallucinations in psychotic disorders. *Schizophrenia Research*, *1*(60), 25–26. doi:10.1016/S0920-9964(03)80074-9

Shawyer, F., Ratcliff, K., Mackinnon, A., Farhall, J., Hayes, S. C., & Copolov, D. (2007). The voices acceptance and action scale (VAAS): Pilot data. *Journal of clinical psychology*, *63*(6), 593–606. doi:10.1002/jclp.20366

Steel, C., Garety, P. A., Freeman, D., Craig, E., Kuipers, E., Bebbington, P., ... & Dunn, G. (2007). The multidimensional measurement of the positive symptoms of psychosis. *International journal of methods in psychiatric research*, *16*(2), 88–96. doi:10.1002/mpr.203

Tein, J. Y., Coxe, S., & Cham, H. (2013). Statistical power to detect the correct number of classes in latent profile analysis. *Structural equation modeling: a multidisciplinary journal*, *20*(4), 640–657. doi:10.1080/10705511.2013.824781

Tolmeijer, E., Hardy, A., Jongeneel, A., Staring, A. B., van der Gaag, M., & van den Berg, D. (2021). Voice-hearers’ beliefs about the causes of their voices. *Psychiatry Research*, *302*(2021), 1–3. doi:10.1016/j.psychres.2021.113997

Vermunt, J. K. (2010). Latent class modeling with covariates: Two improved three-step approaches. *Political analysis*, *18*(4), 450–469. doi:10.2307/25792024

Wongpakaran, N., DeMaranville, J., & Wongpakaran, T. (2021). Validation of the relationships questionnaire (RQ) against the experience of close relationship-revised questionnaire in a clinical psychiatric sample. *Healthcare*, *9*(9), 1–13. doi:10.3390/healthcare9091174

Woods, A., Jones, N., Alderson-Day, B., Callard, F., & Fernyhough, C. (2015). Experiences of hearing voices: analysis of a novel phenomenological survey. *The Lancet Psychiatry*, *2*(4), 323–331. doi:10.1016/S2215-0366(15)00006-1

Yan, T., Fricker, S., & Tsai, S. (2020). Response burden: What is it and what predicts it?. In P. C. Beatty, D. Collins, L. Kaye, J-L. Padilla, G. B. Willis, & A. Wilmot (Eds.) Advances in Questionnaire Design, Development, Evaluation and Testing (Vol 1, pp. 193–212). Wiley & Sons.
